# Improved Climate Risk Simulations for Rice in Arid Environments

**DOI:** 10.1371/journal.pone.0118114

**Published:** 2015-03-16

**Authors:** Pepijn A. J. van Oort, Michiel E. de Vries, Hiroe Yoshida, Kazuki Saito

**Affiliations:** 1 Africa Rice Center (AfricaRice), 01 BP 2031 Cotonou, Benin; 2 Crop & Weed Ecology Group, Centre for Crop Systems Analysis, Wageningen University, P.O. Box 430, 6700 AK, Wageningen, The Netherlands; 3 J. Joordens' Zaadhandel B.V., P.O. Box 7823, 5995 ZG Kessel, The Netherlands; 4 National Agriculture and Food Research Organization, 305-8666 Tsukuba, Japan; University of Minnesota, UNITED STATES

## Abstract

We integrated recent research on cardinal temperatures for phenology and early leaf growth, spikelet formation, early morning flowering, transpirational cooling, and heat- and cold-induced sterility into an existing to crop growth model ORYZA2000. We compared for an arid environment observed potential yields with yields simulated with default ORYZA2000, with modified subversions of ORYZA2000 and with ORYZA_S, a model developed for the region of interest in the 1990s. Rice variety ‘IR64’ was sown monthly 15-times in a row in two locations in Senegal. The Senegal River Valley is located in the Sahel, near the Sahara desert with extreme temperatures during day and night. The existing subroutines underestimated cold stress and overestimated heat stress. Forcing the model to use observed spikelet number and phenology and replacing the existing heat and cold subroutines improved accuracy of yield simulation from EF = −0.32 to EF =0.70 (EF is modelling efficiency). The main causes of improved accuracy were that the new model subversions take into account transpirational cooling (which is high in arid environments) and early morning flowering for heat sterility, and minimum rather than average temperature for cold sterility. Simulations were less accurate when also spikelet number and phenology were simulated. Model efficiency was 0.14 with new heat and cold routines and improved to 0.48 when using new cardinal temperatures for phenology and early leaf growth. The new adapted subversion of ORYZA2000 offers a powerful analytic tool for climate change impact assessment and cropping calendar optimisation in arid regions.

## Introduction

Heat and cold sterility may limit rice production in the current and future climate. Together with phenology they determine in which period rice can be grown with acceptable yield. Crop models can be used to explore options for larger areas and future climates. The simulations by Matthews et al. [1,2] showed large future yield reductions due to increased heat sterility for several regions in parts Asia. Two recent global studies [3,4] show for arid regions such as the Sahel and Pakistan very different impacts of climate change. In the Gourdji paper [3] the arid regions colour dark red showing large climate risks. The Texeira study [4] shows much smaller climate risks in the same regions. We cannot discuss causes of these different outcomes here, but the large discrepancies clearly show that large uncertainty exists in climate change impacts on rice production in arid regions. A lot of experimental research has been conducted on heat and cold sterility risks in rice over the past decade which has not yet been incorporated into existing crop growth models. This study focusses on the ability of the ORYZA2000 model to simulate yields in two sites in Senegal. Senegal is a country with access to river irrigation water, in the Sahel, close to the Sahara desert. Radiation levels are high, humidity is low, temperatures are often above 40°C for days in a row and dangerously low night temperatures (<15°C) occur during part of the year. The large temperature differences within days and at different times of the year make the Sahel regions an interesting site for model evaluation under a wide range of temperature conditions.

As we will show in this paper, the original ORYZA2000 model could not accurately simulate yields in environments with extreme temperatures. We proposed and tested a series of model improvements based on recent experimental research. In environments without severe heat and cold sterility and when phenology is separately calibrated for experiments, ORYZA2000 has been shown to accurately simulate yields [5–15]. On the other hand, it has been shown that the predecessor of ORYZA2000, the ORYZA1 model [16], could not well simulate yields in arid regions such as the Sahel. Main problems identified were poor simulation of phenology, heat and cold sterility. A separate version of ORYZA1, called ORYZA_S, was developed that resolved those issues [17,18]. It included a new submodel for rice development and sterility (RIDEV). Over the last two decades ORYZA1 was extended with ability to simulate water and nitrogen limited production [8]. New research has led to better insights in heat and cold sterility [19,20] in arid and humid climates. These new insights have been incorporated in RIDEV2 (the successor of RIDEV) and they are currently being incorporated into the SAMARA model (Dingkuhn, pers. comm.). At this stage we could have proceeded with either ORYZA_S or with ORYZA2000. We chose to proceed with the ORYZA2000 because we hope that any improvement in the main model can also be useful under other conditions than those tested here, i.e. with water or nitrogen limitation [8] or in crop rotations [21]. With ORYZA_S such applications would not be possible. All parts of the model ORYZA2000 are well documented in a book [8]. The fact that the source code of the ORYZA2000 model is freely available makes the model easily amenable for possible improvements.

Four bodies of experimental research seemed especially relevant for incorporation into the existing model: (1) cardinal temperatures for phenology, (2) cardinal temperatures for early leaf growth, (3) spikelet formation and (4) heat and cold sterility. Firstly, we revisited assumptions on cardinal temperatures. Van Oort et al. [22] showed that phenology simulation could be improved by assuming, for the variety considered in this paper, cardinal temperatures quite different from the default cardinal temperatures in ORYZA2000: a higher base temperature (14°C vs 8°C), a slightly higher optimum temperature (31°C vs 30°C) and no delay in development above the optimum temperature. Secondly, cardinal temperatures are also relevant for simulating early leaf growth, which is generally assumed to be temperature dependent. Under the default settings ORYZA2000 simulates early leaf growth assuming cardinal temperatures of 8, 30 and 42°C, i.e. no leaf growth below 8°C and above 42°C and highest growth rates at 30°C. Most publications on early leaf growth in tropical environments suggest a higher base temperature: Nishiyama et al. [23]: 7–16°C (same data are cited in [24]); Rebolledo et al. [25]: 12°C; Sanchez et al. [26]: 11.8°C for leaf initiation and 14.5°C for shoot growth). Thirdly, we revisited assumptions on modelling spikelet formation. In ORYZA2000, spikelet number is simulated as the total biomass growth from panicle initiation to flowering multiplied with a constant spikelet growth factor (SPGF). Recent work by Yoshida et al. [27] and Kato and Katsura [28] suggested to split up the phase into two separate phases. First, up to about two weeks after panicle initiation juvenile spikelets are formed. The number of juvenile spikelets depends on total N in the crop (kg N ha^−1^). Next in the approximately 2 weeks before flowering, spikelets may be aborted if biomass growth during that period is insufficient. Fourth, we revisited assumptions on heat and cold sterility modelling. In the current ORYZA2000 model, sterility is simulated using daily maximum temperature (T_max_), which generally occurs around 2pm (2 hours after the sun reached its highest point above the horizon). In reality rice more often flowers late in the morning (when temperatures are lower), an adaptive capacity to avoid heat stress [19,29,30]. Another problem with using T_max_ is that it ignores transpirational cooling. Several studies have shown that in arid climates significant transpirational cooling of the panicle can occur [20,31,32, 33,34], leading to panicle temperatures up to 7°C cooler than air temperature at flowering time. Ignoring flowering time and transpirational cooling could lead to overestimation of heat sterility, especially in arid environments. ORYZA2000 simulates cold sterility with a cooling degree days approach in which a temperature sum is accumulated for days with average temperature below a threshold temperature [35,36]. A possible drawback of this method is that in environments with a large diurnal temperature amplitude, such as in the arid climate considered in this paper, average temperature may seem “safe” while in reality the low night temperatures have a severe impact. A second critique on the cooling degree days approach is that it uses air temperature, while using a combination of water and air temperatures could result in more accurate predictions [20,37,38]. While this is well accepted, the challenge for modellers is to how to model water temperature. We used the empirical equations presented in [39,40]. We are aware that more mechanistic water temperature models exist [41,42]. But since we were unsure about their validity in the environment considered here whereas the RIDEV2 model was developed based on observations in the same environment as in which our experiments were conducted, we chose to proceed with the RIDEV2 equation for water temperature.

The objectives of this paper are (1) to integrate above mentioned research into the framework of the ORYZA2000 model and (2) to test systematically for each of them individually and combined how much they contribute to increased accuracy in yield simulation.

## Methods

In the following sections we will describe the models used and the modifications made (§2.1 and §2.2), the experimental data (§2.3) and the methods of model comparison (§2.4)

### ORYZA_S

Although we chose to proceed with ORYZA2000, we did use the ORYZA_S model [17,18] as a benchmark. For this we used the version of ORYZA_S used in a WARDA training course in march 1999. This version contained locally optimised parameters for variety IR64, the same as used in this study. We performed two simulations:

1. With phenology and spikelet number forced to observed values. The method for forcing of phenology is the same as described in section 2.2.1
2. With phenology simulated using RIDEV. Spikelet number in ORYZA_S is simulated using the same method as in ORYZA2000, section 2.2.3 of this paper

ORYZA_S uses the RIDEV model for simulating phenology, a modification relative to the ORYZA1 and ORYZA2000 method of simulating phenology. For RIDEV, we used the IR64 parameter values as present in the ORYZA_S code. These parameters were determined in the early 1990s based on similar experiments as described in this paper. ORYZA_S uses modified heat and cold sterility equations based on Dingkuhn and Sow [17,18]. Cold sterility is calculated based on the average of minimum air temperatures in the period from development stage 0.85 to 1.0, approximately the 14 days before flowering. Heat sterility is calculated based on daily average temperatures, averaged over the period from development stage 0.95 to 1.25, approximately the 12 days centred around the 50% flowering date.

### ORYZA2000 with modifications

Our starting point was the ORYZA2000 model version 2 number 13. Subversions of the model were developed incorporating theories described in the following subsections. For naming convention, we suggest to use ORYZA2000v2n13s1 (version 2, number 13, subversion 1), which we will for brevity in this paper refer to as s1 to s26. All acronyms used in the sections below are listed in a supporting table (S1 Table).

The ORYZA2000 model dynamically simulates physiological processes in interaction with their environment [8]. The ORYZA2000 has been extensively validated in other studies (see the list of references cited in the introduction). We used default crop parameters from the crop file for variety IR72, which provide a good base assumption for other high yielding irrigated lowland varieties such as IR64 [15]. Any modifications to parameter values specifically for IR64 are discussed in the following sub sections. We simulate potential production, which is production free from weeds, pests, diseases, water stress and nutrient stress. It is impossible to describe the full model here but a good book and website are available [8], https://sites.google.com/a/irri.org/oryza2000/home). In the following section we document the modifications made.

### Phenology

In ORYZA2000 the following developmental phases are discerned:

- Basic Vegetative Phase (BVP): DVS = 0 to DVS 0.4
- Photoperiod Sensitive Phase (PSP), DVS 0.4 to 0.65, ending at panicle initiation
- Post PSP phase (PPP), DVS 0.65 to 1.0, ending at 50% flowering
- Grain Filling Phase (GFP), DVS 1.0 to 2.0, ending at maturity

We applied two approaches for simulating phenology (motivation for this is in §2.4). In step 1 we forced phenology to observed values using the very simple approach below. Daily development rates (unit d^−1^) were set to:

$${\mathrm{DVR}}_{\mathrm{BVP},i}={\mathrm{DVR}}_{\mathrm{PSP},i}=0.65/\left({\mathrm{OBSDUR}}_{\mathrm{BVP},i}+{\mathrm{OBSDUR}}_{\mathrm{PSP},i}\right)$$ (1)

$${\mathrm{DVR}}_{\mathrm{PPP},i}=\left(1.0-0.65\right)/{\mathrm{OBSDUR}}_{\mathrm{PPP},i}$$ (2)

$${\mathrm{DVR}}_{\mathrm{GFP},i}=\left(2.0-1.0\right)/{\mathrm{OBSDUR}}_{\mathrm{GFP},i}$$ (3)

Where OBSDUR_p,i_ is the duration of phase p in treatment i in days. Note that the point of transition from the BVP to PSP was not observed, therefore these phases were combined. Development stage DVS was then calculated adding up these respective development rates, accumulating one DVR unit per day. This approach ensures exact reproduction of observed dates of emergence, panicle initiation, flowering and maturity without having to go through the complexities of separately calculating temperature sum dependent development rates for each phase and each sowing date.

In step 2 we simulated phenology using the bilinear temperature response model and hourly air temperatures. We created one model subsversion with default parameters: cardinal temperatures (8, 30, 42°C for base, optimum and maximum temperature for development), no photoperiod sensitivity and a transplanting shock parameter of 0.4. We also created subversions with optimised cardinal temperatures (14, 31, 999°C), no photoperiod sensitivity and a transplanting shock parameter of 0.0. These parameters were previously determined from the same phenological and climate data as used in this paper, using a phenology calibration program described in [22]. This phenology calibration program calibrates all phenological parameters simultaneously and minimises correlation between phenology errors and temperature. Basically this research showed that with the default cardinal temperatures, duration from emergence to flowering was underestimated at lower temperatures and overestimated at higher temperatures. We will present further on in the paper a comparison of simulated duration from emergence to flowering as obtained with these different cardinal temperatures. The base and optimum temperatures obtained by van Oort et al. [22] are consistent with a recent independent review of base, optimum and maximum temperatures for development [26]. Note that these base temperatures are for a bilinear temperature response model. Other studies have shown that phenology can be more accurately simulated with a sigmoid or bell shaped function [43,44]. In such functions we often find lower base temperatures but with hardly any increase in development rate (low slope) up to somewhere between 10 and 16°C. So the meaning of the base temperature is different depending on the model used. In practice nobody would grow rice under temperatures close to the base temperature for prolonged time, in that sense this base temperature will always remain somewhat hypothetical. Likewise uncertainty exists in the nature of temperature response above the optimum temperature [45], The SIMRIW model and cardinal temperatures calibrated by van Oort et al. [22] suggest no decrease in development while several other phenology models show a sharp decrease in development towards a maximum temperature. Few studies have been conducted in environments with temperatures above the optimum for a long time. The Sahel environment is interesting for phenological studies because such contrasting temperatures occur, often for prolonged times above the optimum temperature for development.

### Leaf growth

Leaf growth in ORYZA2000 is split into two phases. When the leaf area index (LAI) is below 1.0 (m^2^ leaf m^−2^ soil) leaf growth is simulated as a function of temperature only. While LAI is above 1.0, leaf growth is simulated as dependent on net photosynthesis and assimilate partitioning to leaves. A mechanism has been implemented in subroutine SUBLAI3 to ensure a smooth transition between temperature and radiation driven LAI growth around the LAI = 1 point. For the first (LAI<1) phase, hourly air temperatures are calculated as [8: p. 31]:

$$T_{\mathrm{air}}\left(t\right)=\frac{\left(T_{\max}-T_{\min}\right)}{2}+\frac{\left(T_{\max}-T_{\min}\right)}{2}\times \cos\left(0.2618\times \left(t-14\right)\right)$$ (4)

Hourly heat units for leaf development (HULV(t), °Ch) are calculated as [8: p. 32]:

$$\mathrm{HULV}\left(t\right)=\left\{ \begin{array}{c} 0\\ T_{\mathrm{air}}\left(t\right)-\mathrm{TBLV}\\ \left(\mathrm{TOD}-\mathrm{TBLV}\right)\left(\frac{\mathrm{TMD}-T_{\mathrm{air}}\left(t\right)}{\mathrm{TMD}-\mathrm{TOD}}\right) \end{array}\begin{array}{c} \;\text{for}\\ \;\text{for}\\ \;\text{for} \end{array}\begin{array}{c} \;T_{\mathrm{air}}\left(t\right)\leq \mathrm{TBLV},\;T_{\mathrm{air}}\left(t\right)\geq \mathrm{TMD}\\ \mathrm{TBLV}<T_{\mathrm{air}}\left(t\right)\leq \mathrm{TOD}\\ \mathrm{TOD}<T_{\mathrm{air}}\left(t\right)\leq \mathrm{TMD} \end{array}\right.$$ (5)

The same approach is used for calculating thermal time for phenology. In these equations TBLV, TOD, and TMD are the base, optimum and maximum temperature for leaf development.

From the hourly values, the daily average heat unit (HULV, °Cd) is calculated. Daily LAI growth, GLAI, is calculated as:

GLAI=LAI×RGRL×HULV (6)

Where RGRL is the “relative growth rate of the leaves” parameter. Equation 6 results in an LAI growing exponentially with temperature sum (= accumulated HULV over multiple days), see Bouman et al. [8: p. 65].

For the phase when LAI is greater than 1, LAI keeps growing depending on simulated leaf mass growth (RWLVG) and specific leaf area (SLA) and loss of leaves dues to senescence (LLV). RWLVG depends on total net biomass growth and on the fraction assimilates partitioned to the leaves FLV. In the model FLV and SLA are both functions of development stage. It was noted during simulations that for the crops with very long periods from emergence to flowering, LAI would reach very high values. This occurred in Fanaye with sowing in October to December and in Ndiaye with sowings from October to April. During these periods low temperatures ensured a long vegetative period while high radiation levels allowed for high LAI growth. The highest simulated LAI was 22. Such LAI values are not realistic. What is more realistic is a maximum of around 10. Even an LAI of 10 will only be obtained under exceptional conditions (long growing season, ample nutrition, water and radiation). The unrealistic high LAI values may have different causes. One could be that the simulations overestimate early growth. We first tried modifying the parameter RGRL. This lead to improved predictions for some sowing dates but poorer prediction for the other dates. Replacing the cardinal temperatures was more effective, and as we showed in the introduction, several studies [23–26] suggested base temperatures higher than the default 8°C for TBLV. A second possible explanation for the unrealistic high LAI is that the default model does not account for reduced assimilate partitioning to the leaves (FLV) at lower temperatures [17,18]. We implemented temperature dependent partitioning as:

$$\mathrm{FLV}\;=\{\begin{array}{ccc} \;0.33\;+\;0.0091\;\times \;\mathrm{TAV} & & 0.0\leq \mathrm{DVS}\leq 0.3\\ 0.48\;-\;0.49\;\times \;\mathrm{DVS}\;+\;0.0091\;\times \;\mathrm{TAV} & & 0.3<\mathrm{DVS}\leq 0.8\\ \;\left(1-\mathrm{DVS}\right)\;\times \;\left(0.45+0.045\times \mathrm{TAV}\right) & & 0.8<\mathrm{DVS}<1.0 \end{array}$$ (7)

$$\mathrm{TAV}\;=\;\left(T_{\max}-T_{\min}\right)/2$$ (8)

With these equations FLV is higher for higher daily average temperatures (TAV, °C). FLV decreases with development stage (DVS) to 0 at flowering (DVS = 1.0). The fraction assimilates partitioned to the storage organ (FSO) is a fixed function of development stage. And the fraction assimilates partitioned to the stem is calculated as 1—FLV—FSO. Under default settings FLV also decreases with DVS [8: p. 172] but FLV is not adjusted by TAV as above.

A third possible explanation for the unrealistic high LAI is that leaves in the bottom of the canopy could die when for prolonged time insufficient radiation penetrates through the canopy and when leaves reach a certain age. This process, which we will refer to as “shading to kill leaves”, is not present in ORYZA2000 version 2 number 3. Following the SUCROS model [46,47] we used the following function to kill leaves:

LLVSH=max(0.0,min(0.03,0.03×(LAI−LAICR)/LAICR)))×WLVG (9)

Where LLVSH (kg DM ha^−1^ d^−1^) is the loss of leaf dry matter (DM) due to shading, WLVG is total green leaf dry matter (kg DM ha^−1^) on a given day and LAICR = 4.0 (m^2^ leaf m^−2^ soil) is the critical LAI above which leaves start to die. According to this equation, no leaves die below LAICR. Above LAICR the fraction of leaves dying increases linearly with LAI, up to a maximum of 3% a day at LAI = 8 and higher. A very similar shading function is used in the WOFOST crop growth model. Version 3 of the ORYZA2000 model also has a “shading to kill leaves” function that accounts for genetic variation in tolerance to shading and which simulates higher death rates at lower radiation levels (Tao Li pers. comm.). We simulated with these different approaches for “shading to kill leaves” and found that they resulted in almost similar values for accuracy of yield simulation. Since we lacked LAI data for validation, it remains impossible to tell which one is most correct. For simplicity we will present only results of the Equation 9 “shading to kill leaves” function.

We created model subversions for early growth (Eq 5–7) by using either (1) the default (8, 30, 42°C) values for TBLV, TOD, and TMD or (2) the values obtained from the phenology calibration exercise discussed in previous section (14, 31, 999°C). We created subversions by using for partitioning either (1) the default non-temperature dependent function or (2) the temperature dependent partitioning function (Eq 8). We created subversions without and with the “shading to kill leaves” function (Eq 9).

### Spikelet formation

In ORYZA2000 and in ORYZA_S the number of spikelets formed during the PPP phase (panicle initiation to flowering) is calculated as [8: p. 60]:

NSP=∑CGR×SPGF (10)

Where GCR is the gross biomass growth in kg dry matter per day simulated by the model. SPGF, the spikelet growth factor, is a parameter indicating the number of spikelets formed per kilogram of dry matter formed (no kg^−1^). Summation is over the PPP development phase (0.65<DVS<1.0). The experimentally determined SPGF is 64900 for variety IR72 [8: p. 61].

Yoshida et al. [27] and Kato and Katsura [28] split up the PPP phase into two separate phases. First, up to about two weeks after panicle initiation, juvenile spikelets are formed. Their number depends linearly on total nitrogen (N) in the crop (kg N ha^−1^). Next in the approximately 2 weeks before flowering, spikelets may be aborted if biomass growth is insufficient. ORYZA2000 simulates the N content in leaves, in g N m^−2^ leaf ha^−1^. From this in combination with specific leaf area (SLA) we can calculate the new variable for leaf N content (NCLV, kg N per kg leaf). Stem N content (NCST) is assumed to be half of leaf N content (cf [8: p. 101]). We multiply these N contents with leaf and stem biomass (kg DM ha^−1^) to obtain total N of the aboveground biomass (NBIOM, kg N ha^−1^). At DVS 0.825, halfway between panicle initiation and flowering, we simulate the total number of juvenile spikelets as:

NSPJUV=NBIOM×SPGFJ (11)

**Fig 1:**
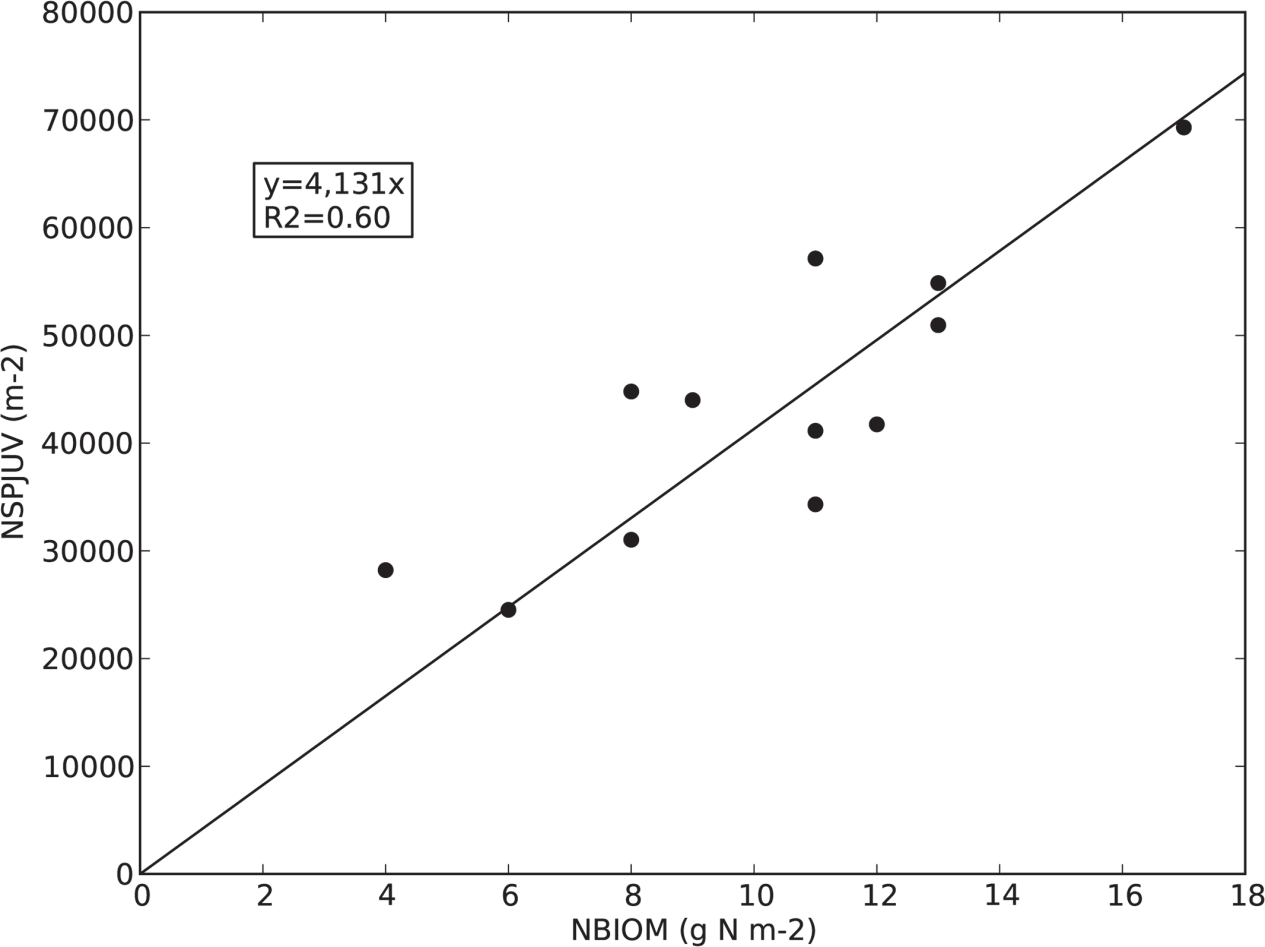
Relation between number of juvenile spikelets as a function of total nitrogen in above ground biomass, variety IR72, based on data presented in Yoshida et al. [27].

Where SPGFJ is the spikelet growth factor for juvenile spikelets formed per kilogram of N in the total above ground biomass. This model is a simplification of the Yoshida et al. [27] model in which at very low NBIOM spikelet formation is higher than expected based on a linear Equation (11). We chose for this simpler model for its simplicity and because NBIOM in our experiments was always expected to be high. Fig. 1 shows for variety IR72 the relation between NSPJUV and NBIOM, with SPGFJ = 4,131,400 juvenile spikelets / kg N (or 4,131 juvenile spikelets per gram N as in Fig. 1). The NSPJUV values shown in this figure were not directly measured, but inferred from final NSP taking into account an abortion function for which the parameter was estimated as described in [27].

In the second phase, a fraction of the juvenile spikelets is aborted (see also [48,49]). We denote the days from DVS 0.825 to flowering (DVS = 1.0) as CNT2BF and the sum of daily gross biomass growth during this period as GCR2BF = ΣGCR. With the Yoshida model [27] the final number of spikelets (no ha^−1^) was calculated at flowering as:

$$\mathrm{NSP}=\mathrm{NSPJUV}\times \left(1-e^{\left(-0.1232\times 0.1\times \frac{\mathrm{GCR}2\mathrm{BF}}{\mathrm{CNT}2\mathrm{BF}}\right)}\right)$$ (12)

Where the exponential term is the fraction aborted spikelets and 0.1 is a unit conversion term. Kato and Katsura [28] proposed an alternative model for abortion:

$$\mathrm{NSP}=\mathrm{NSPJUV}\times \left(1-e^{\left(-0.61\times \frac{\mathrm{GCR}2\mathrm{BF}}{\mathrm{NSPJUV}\times 3.0\times 10^{-6}}\right)}\right)$$ (13)

In this equation NSPJUV is multiplied with floret weight, which we assumed to be 3 mg per floret.

Broadly, the three approaches are similar in that more biomass growth from PI to flowering results in more spikelets. Under N limitation, the Yoshida model [27] and the Kato and Katsura model [28] predict lower spikelet formation. But also the default Equation 10 would predict less spikelet formation under N stress, as N stress would also reduce GCR. Also under non-limiting conditions such as in our experiments the three approaches can give different results, due to their sensitivity to the leaf mass: stem mass ratio. Imagine two crops with the same total leaf + stem mass, but one crop has relatively more leaves. Since N content of the leaves is higher than that of the stems, the crop with more leaves will produce more juvenile spikelets (Eq 11). Abortion would also be lower if the higher leaf mass results in a higher GCR2BF. Together, production of mature spikelets (NSP) would be higher. We may therefore expect that the accuracy of the spikelet formation calculation depends strongly on accurate simulation of leaf and stem growth. We compared the three approaches for simulating spikelet number, using either the default value for SPGF (= 64900) or SPGFJ = 4131400 from Fig. 1.

### Heat fertility

Heat fertility in ORYZA2000 is simulated as a function of maximum air temperature [8: p. 61] based on earlier work by Horie [36]:

$$\overset{-}{SFHEAT}\;=\;\frac{1}{1+\exp\left(0.853{\times T}_{m,a}-36.6\right)}$$ 14

Where *T*_*m*,*a*_ is the average of maximum air temperatures from DVS 0.96 to 1.2, a period of approximately 10 days centred around the 50% flowering date. Equation 14 describes a sigmoid with 90% fertility at 34°C, 50% fertility at 36.6°C and 10% fertility at 39.2°C.

Our new heat sterility model is based on recent work by Julia and Dingkuhn [19,20], van Oort et al. [32] and Jagadish et al. [50], but inspired also by works of Matsui et al. [31] and Weerakoon et al. [51] who showed the importance of correcting for transpirational cooling. Improvements relative to the ORYZA2000 model include consideration of flowering time within the day (earlier flowering leads to exposure to lower temperatures) and transpirational cooling. First, peak flowering time was calculated based on the average of minimum temperatures in the preceding 7 days (*T*_*min7*_) and sunrise time (*t*_*sunrise*_), a function from Julia and Dingkuhn [17]:

$$t_{\mathrm{peakfl}}=t_{\mathrm{sunrise}}+12.7-0.348\times T_{\min7}$$ (15)

Air temperature at peak flowering time was calculated by using the Goudriaan and van Laar [52] and Ephrath et al. [53] diurnal temperature model, with *T*_*min*_ and *T*_*max*_ the minimum and maximum air temperature and *DL* the daylength (see also [32]):

$$T_{\mathrm{air}}\left(t_{\mathrm{peakfl}}\right)=T_{\min}+\left(T_{\max}-T_{\min}\right)\times \text{sin}\;\left(\pi \times \frac{t_{\mathrm{peakfl}}-t_{\mathrm{sunrise}}}{\mathrm{DL}+2*1.5}\right)$$ (16)

The vapour pressure deficit *VPD* (in kPa) was calculated from the early morning vapour pressure VPA (which was calculated assuming *T*_*dew*_ *= T*_*min*_, see §2.3.2) and the saturated vapour pressure at *T*_*air*_(*t*_*peakfl*_), Equation 17. Panicle temperature *T*_*pan*_(*t*_*peakfl*_), was then calculated based on the equation reported in Julia and Dingkuhn [18]:

$$\mathrm{VPD}\left(t_{\mathrm{peakfl}}\right)=0.6107\times \left(\text{exp}\;\left(\frac{T_{\mathrm{air}}\left(t_{\mathrm{peakfl}}\right)\times 17.4}{239+T_{\mathrm{air}}\left(t_{\mathrm{peakfl}}\right)}\right)-\text{VPA}\right)$$ (17)

$$T_{\mathrm{pan}}\left(t_{\mathrm{peakfl}}\right)=T_{\mathrm{air}}\left(t_{\mathrm{peakfl}}\right)-1.29\times \mathrm{VPD}\left(t_{\mathrm{peakfl}}\right)-0.01$$ (18)

Transpirational cooling with Equation 18 can be as large as 7°C (at *RH* = 25%, *T*_*air*_ = 40°C). A second empirical model for transpirational cooling was recently published [32]. Within the range of relative humidities and temperatures present in our environment this second model predicts similar magnitudes of transpirational cooling. Since the choice between the two is then arbitrary, but the Julia and Dingkuhn [17] empirical model was based on a larger dataset, we chose to simulate only with their model.

Different equations have been presented for the relation between panicle temperature and heat fertility. Some of these are based on counts of germinated pollen or counts of pollen on stigma (for example see [31,51, 54–58]). Others are based on the assumption that the fraction filled spikelets (FFS) is the same as the fertility (e.g. [59,60,61]). The latter assumption is valid only when the crop is sink limited, under source limitation FFS will be less than fertility (SPFERT). Especially under favourable conditions, rice can produce more spikelets than it can ever fill [49] leading to source limitation and hence reduced FFS values. Source limitation is also more likely when drought stress occurs, resulting in a lower FFS value [62]. Due to difficulties in quantification of source size, some doubts may always remain on the validity of the FFS = SPFERT assumption. For these reasons, we preferred to use an equation with pollination based parameters. The following equation was derived specifically for variety IR64 and based on measured spikelet temperatures [50]:

$$\mathrm{SFHEAT}=\frac{\text{exp}\left(14.3-0.408*T_{\mathrm{pan}}\left(t_{\mathrm{peakfl}}\right)\right)}{1.0+\text{exp}\left(14.3-0.408*T_{\mathrm{pan}}\left(t_{\mathrm{peakfl}}\right)\right)}$$ (19)

In this sigmoid 90% of the spikelets are still fertile at 29.7°C, 50% of the spikelets are fertile at 35.0°C and 10% of the spikelets are fertile at 40.0°C. With a sigmoid fertility decreases more and more per unit (°C) temperature increase up to the 50% fertility point. Beyond this point, fertility increases less and less per unit (°C) temperature increase. More realistic might be a continuously increasing sensitivity [51]. But as in our data we never came across the 50% point and Equation 19 was determined particularly for IR64, the variety used in our simulations, we proceeded with this equation. Acknowledging that flowering occurs over a number of days we counted the days from DVS 0.96 and DVS 1.2 (SIMDUR_0.96–1.2_) and averaged daily *SFHEAT* values over this period:

$$\bar{\mathrm{SFHEAT}}=\mathrm{SFHEAT}/{\mathrm{SIMDUR}}_{\;0.96-1.2}$$ (20)

### Cold fertility

Cold fertility in ORYZA2000 is simulated as a function of cold temperature sum *COLDTT*, accumulated from DVS 0.75 to 1.2 when daily average temperature TAV (calculated as (T_max_—T_min_)/2)) is below 22°C:

COLDTT=∑(22−TAV) (21)

$$\mathrm{SFCOLD}=1-\left(4.6+0.054\times {\mathrm{COLDTT}}^{1.56}\right)/100$$ (22)

Our new method for simulating cold fertility considers two cold sensitive phases [18]. The microspore stage starts at panicle initiation and lasts till approximately halfway flowering. The panicle exsertion stage starts at the end of the microspore stage and continues till flowering. According to Julia and Dingkuhn [18] cold during the microspore stage causes sterility and cold during the panicle exsertion stage causes incomplete exsertion of the panicle. Moreover non exserted spikelets are also infertile. During these two stages the meristem moves up in the canopy from being at flood level at panicle initiation to in the top of the canopy at flowering. Therefore, it would be best to simulate using flood water temperature as input at panicle initiation and air temperature as input at flowering, with in between a weighted average depending on crop stage [37,38]. ORYZA2000 does not simulate panicle position within the canopy. As a simplification we therefore chose to simulate microspore fertility (SFCOLD1) with minimum floodwater temperature and panicle exsertion stage fertility (SFCOLD2) with minimum air temperature. We assumed the transition between these two phases was at DVS 0.825, halfway between panicle initiation (DVS 0.65) and flowering (DVS 1.0). *SFCOLD*1 was simulated as a function of minimum floodwater temperature, which depends on how much radiation reaches the floodwater and diurnal air temperature amplitude. Radiation reaching the floodwater depends on light transmission through the canopy. ORYZA2000 simulates the leaf area index (LAI). We simulated the light transmission ratio (LTR) assuming an extinction coefficient of k = 0.6 [39,40]:

LTR=exp(−0.6×LAI) (23)

Minimum floodwater temperature (T_w,min_) was calculated based on an empirical relation derived based on thousands of observations across a wide range of environments [39,40]:

$$\begin{array}{l} T_{w,\min}\;=T_{\min}+0.658\times \left(T_{\max}-T_{\min}\right)\times \left(1-LTR\right)+\\ \;0.425\times \left(T_{\max}-T_{\min}\right)\times LTR-0.303\times {\left(T_{\max}-T_{\min}\right)}^{1.2} \end{array}$$ (24)

This model (as is any model) is a simplification, it does not account for floodwater depth, turbidity, temperature of inflowing water etcetera. At a later stage, incorporation of more sophisticated models [41,42] may be considered. Julia and Dingkuhn [18] described the following parabolic relation for cold sterility during the microspore stage:

$$\mathrm{SFCOLD}1=\text{max}\left(0,1-\left(0.0094\times T_{w,\min}^{2}-0.431\times T_{w,\min}+\;5.039\right)\right)$$ (25)

Two conceptual objections against this equation are that *SFCOLD*1 can never get bigger than 0.9 (90%) and that cold sterility decreases again above *T*_*w*,*min*_ = 23°C. Julia and Dingkuhn [18] assumed fertility was equal to the fraction filled spikelets, FFS. Here we do want to allow our model to reach 100% fertility and we presume the remaining 10% unfilled may have been caused by source limitation. We therefore fitted the following linear model through the data of Julia and Dingkuhn ([18], Fig. 7 of their paper):

$$\mathrm{SFCOLD}1=\text{min}\left(1,\text{max}\left(0,1-(20-T_{w,\min}\right)/\left(20-13\right)\right))$$ (26)

In this model fertility is 1 for T_w,min_ > 20°C and decreases linearly to 0 at 13°C. Since the microspore stage lasts approximately 14 days, the question is how to calculate aggregate $\bar{\mathrm{SFCOLD}1}$ over this period. We consider two alternative rules for this:

$$\bar{\mathrm{SFCOLD}1}=\mathrm{SFCOLD}1/{\mathrm{SIMDUR}\;}_{0.65-0.825}\;\left(\text{averaging rule}\right)$$ (27)

$$\bar{\mathrm{SFCOLD}1}=\text{min}\left(\mathrm{SFCOLD}1\right)\;\left(\text{minimum rule}\right)$$ (28)

Where either the average or minimum over the duration (in days) of the microspore stage (0.65<DVS<0.825) is taken. The approaches can have a different effect when one or few very cold days happen to occur during the microspore stage, in that case the minimum rule will simulate lower SFCOLD1 than the averaging rule. Such conditions occur when the microspore stage happens to occur at the start or at the end of the coldest part of the year. Such conditions occurred for emergence months 9 and 11 (September and November) in our experiments. We therefore simulated with both approaches and compared their effect on accuracy of yield simulation.

For the panicle exsertion phase we assumed the same fertility function (with 20 and 13°C) but calculated daily fertility using minimum air temperature:

$$\mathrm{SFCOLD}2=\text{min}\left(1,\text{max}\left(0,1-\left(20-T_{\min}\right)/\left(20-13\right)\right)\right)$$ (29)

Again, discussion may arise on how to calculate aggregate SFCOLD2. Since panicle exsertion is a more continuous processes in which slow exsertion in one day can be compensated by faster exsertion on another day we chose to calculate the aggregate SFCOLD2 as the average of daily SFCOLD2 values over the days from DVS 0.825 to DVS 1.0:

$$\bar{\mathrm{SFCOLD}2}=\mathrm{SFCOLD}2/{\mathrm{SIMDUR}\;}_{0.825-1.0}$$ (30)

Another issue is whether $\bar{\mathrm{SFCOLD}2}$ should be combined with $\bar{\mathrm{SFCOLD}1}$ to calculate final cold fertility, or whether $\bar{\mathrm{SFCOLD}2}$ should be multiplied with spikelet number. Practically for yield simulation and validation this will have no impact. But it does matter when we validate and calibrate spikelet formation. In that case, one should take care of having consistency in definitions. It would be wrong if the model output showed only the exserted spikelets while observations are the sum of exserted and non-exserted spikelets. In our experiment, spikelets observed are only the ones exserted. Non-exserted spikelets were not counted. Therefore for consistency we multiplied the number of mature spikelets (NSP) with $\bar{\mathrm{SFCOLD}2}$ and calculated SFCOLD as $\bar{\mathrm{SFCOLD}1}$.

### Combined fertility and effect on yield

Combined fertility in ORYZA2000 is calculated as SPFERT = min(SFHEAT, SFCOLD). We proceeded with the same rule substituting SFCOLD (Eq 22) with $\bar{\mathrm{SFCOLD}1}$ (Eq 27 or 28) and SFHEAT (Eq 14) with $\bar{\mathrm{SFHEAT}}$ (Eq 20). Again, discussion may arise how to calculate aggregate fertility. We therefore also calculated combined fertility as SPFERT = SFHEAT x SFCOLD (product rule). In many cases the distinction between these rules may seem trivial, as it will be very rare to find cold sterility during the microspore stage followed by heat sterility in the flowering stage. In normal cases SFHEAT will equal 1 if SFCOLD is less than 1 and vice versa. Again, with the large diurnal temperature amplitudes of our dataset, it may become an issue. SPFERT sets the upperbound for the fraction filled spikelets (FFS) and for the simulated yield. Yield in the model output is called WRR (weight rough rice, kg DM ha^−1^). From SPFERT we calculate the highest possible WRR as:

PWRR=NSP×SPFERT×WGRMX (31)

Where NSP is the number of spikelets per hectare (see §2.2.3) and WGRMX is the maximum grain weight which we assumed to be 25 mg per grain. After flowering ORYZA2000 simulates grain filling based on assimilation and respiration, which in turn depend on environmental conditions, amount of stem reserves, LAI at flowering, senescence and duration of the grain filling phase. When the crop is sink limited (if NSP or SPFERT are low) WRR will not become larger than PWRR. Any excess assimilates produced are in such cases partitioned to the stems.

### Experimental data

#### Sites and crop data

We used experimental data previously reported in de Vries et al. [59], for variety IR64. The crop was sown once per month at two sites in Senegal, for 15 sowing dates in a row in the years 2006–2007, thus there were 30 treatments. The site Ndiaye (16°11’N, 16°15’W) is located in the Senegal river delta, the site Fanaye (16°32’N, 15°11’W) is located along the same river approximately 150 km inland. The weather is more extreme in this site. Dates of emergence, panicle initiation, 50% flowering and maturity, spikelet number, grain weight and fraction filled spikelets were recorded. We took averages of observed values from 3 replicates for each treatment. The crops were well fertilised and kept free from weeds. Diseases were not a problem. With 3 replicates per treatment and 30 treatments it was practically and economically not feasible to sample within the growing season. Consequentially calibration and validation of LAI, organ biomass and organ N content during the growing season was not possible. No crop data other than developmental stages (phenology) were recorded for the 18 December emergence date in Ndiaye. Thus we have n = 29 treatments.

#### Weather data

**Fig 2:**
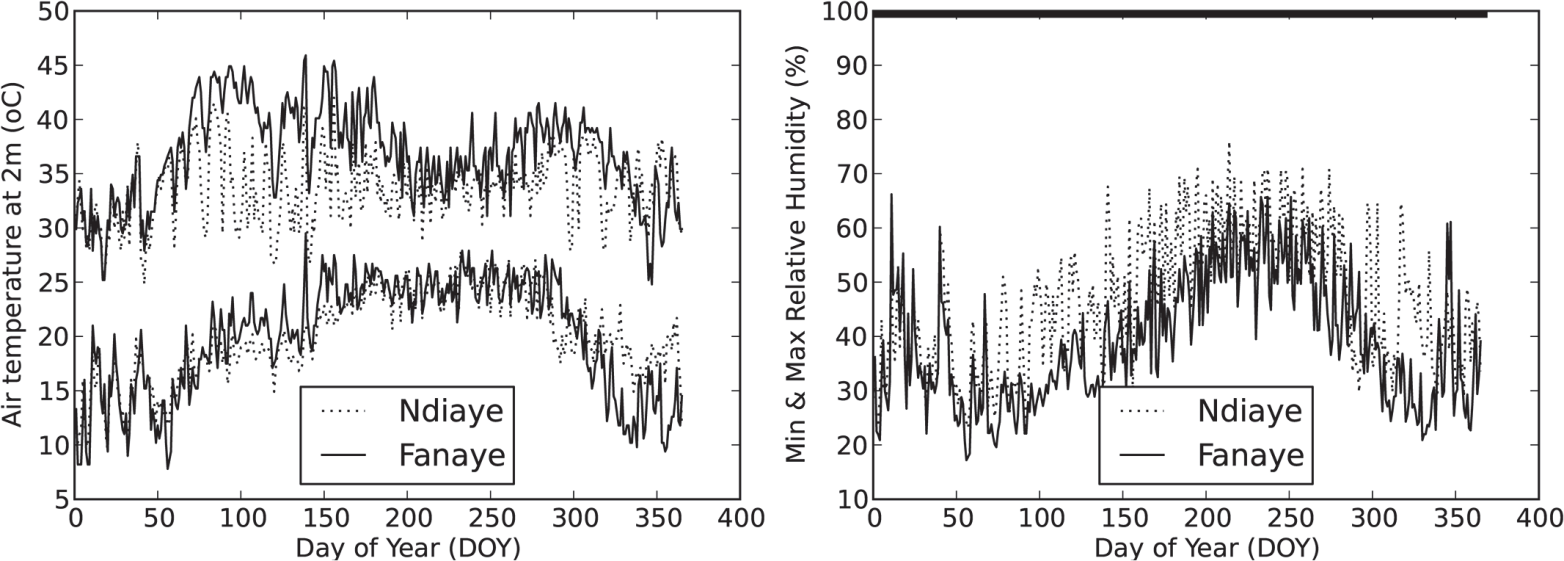
Daily minimum and maximum temperature (left) and minimum relative humidity (right) in the two sites (RHmax is assumed to be 100%).

Minimum and maximum air temperatures were measured from stations located in a rice field adjacent to the experiment. Wind speed and precipitation, dew point temperature and radiation were recorded, but instruments for dew point temperature and radiation failed during part of the season, caused by a storm after which replacement took a long time. In Fanaye, the weather station failed altogether in 2007, for that year we replaced temperatures with those observed for the same dates in 2006. We chose to replace all radiation with radiation from the NASA POWER database. It has been shown that this satellite based dataset provides unbiased and quite accurate radiation data when compared with radiation observed on the ground [63]. The ORYA2000 adjusts maximum photosynthesis rate according to atmospheric CO2 concentration [8: p. 48]. For atmospheric CO2 concentration we assumed a value of 382 ppm (taken from the Mona Lau record) which is representative for the period in which the experiments were conducted. Since water was non-limiting during the growing season, missing precipitation did not affect simulations. For simulation of heat sterility and transpirational cooling we needed to know the actual vapour pressure. We assumed dew point temperatures (T_dew_) equal to minimum air temperatures, which results in RH_max_ being 100% early in the morning. This is a fair assumption for rice cultivated in continuously flooded paddy fields. The resulting annual pattern of RH_min_ (Fig. 2, bottom) is similar to that reported in Dingkuhn et al. [60] for the same environment. No soil data were used for the simulations, as these are also not needed for simulating potential production. The problems with weather data are a common problem in much of the experimental research ongoing in developing countries. Good weather stations are expensive and replacement can take a long time. Weather logs may not be sent automatically to a central database and as a result malfunctioning may not be immediately detected. The 2007 missing data for Fanaye in combination with reported growing periods will have an effect on 7 out of 30 sowing dates. We compared accuracies for the first 8 and last 7 dates at Fanaye. Accuracies were not structurally different for the two periods.

### Accuracy assessment and comparison

Ideally the proposed modifications in heat and cold sterility should translate into improved yield predictions. However if other components of the model (phenology, leaf growth, spikelet formation) are also inaccurate then effects of modifications in heat and cold sterility may remain obscured. For this reason we validated the model in two steps:

Step 1: simulation of yields with observed phenology and observed spikelet number
Step 2: simulation of yields with simulated phenology and simulated spikelet number

Forcing phenology and spikelet number to observed values allows us to assess accuracy improvement obtained with the different heat and cold sterility models, without results being affected too much by other possible errors. It is useful for this purpose, but in the end, one wants to know how accurate the model is if all processes are actually simulated. Therefore in step 2, we put the model to a more rigorous test by simulating all processes and with a minimum of additional calibration.

Within step 1 and 2, a series of model subversions (s1 to s26) were constructed. Each subversion was used to simulate yields for each of the n = 29 treatments. By changing one process at a time in the different subversions we could clearly retrace causes of increases in accuracy. For each subversion, goodness of fit was calculated as modelling efficiency [64]:

$$EF=1-\frac{\sum {\left(S_{i}-O_{i}\right)}^{2}}{\sum {\left(\bar{O}-O_{i}\right)}^{2}}$$ (32)

Where *S*_*i*_ is simulated Yield in treatment *i*, *O*_*i*_ is observed Yield in treatment *i* and *Ō* is the average of observed yields. A value of 1 for EF indicates perfect prediction. A value of 0 means the model predicts no better than when we would simply take the mean of observations. Negative values suggest that the average of observed values is a better predictor than the model.

One way of looking at the data is through metrics such as EF. Another way of looking at the data is through visual comparison. It is possible that the model simulates the general pattern well, but is shifted away from observed data. For example the model may be systematically a bit too high because simulated potential yields are not so easily practically achieved, but may properly simulate the direction in which yields change with different sowing dates. Or yields may dramatically drop within a short timespan, making the model very sensitive to relatively small errors in phenology simulations and observations. In such cases EF can be less than 1 but visually we might still judge the model as having a good fit. We therefore also present graphics of simulated and observed yields at different sowing dates.

## Results

Fig. 2 shows the temperatures and humidity in the two sites through the year. Weather is more extreme in Fanaye. Maximum temperatures in Fanaye are consistently higher but especially so during days 75 to 200. From day 320 to 365, the minimum temperatures in Fanaye are lower than in Ndiaye. Relative humidity is lower in Fanaye. Such patterns are consistent with the more inland position of the Fanaye site. More details on weather data and site can be found in de Vries et al. [59,65] and in Dingkuhn et al. [17,18,60] for earlier years in the same two sites.

### With observed phenology and spikelet number

**Fig 3:**
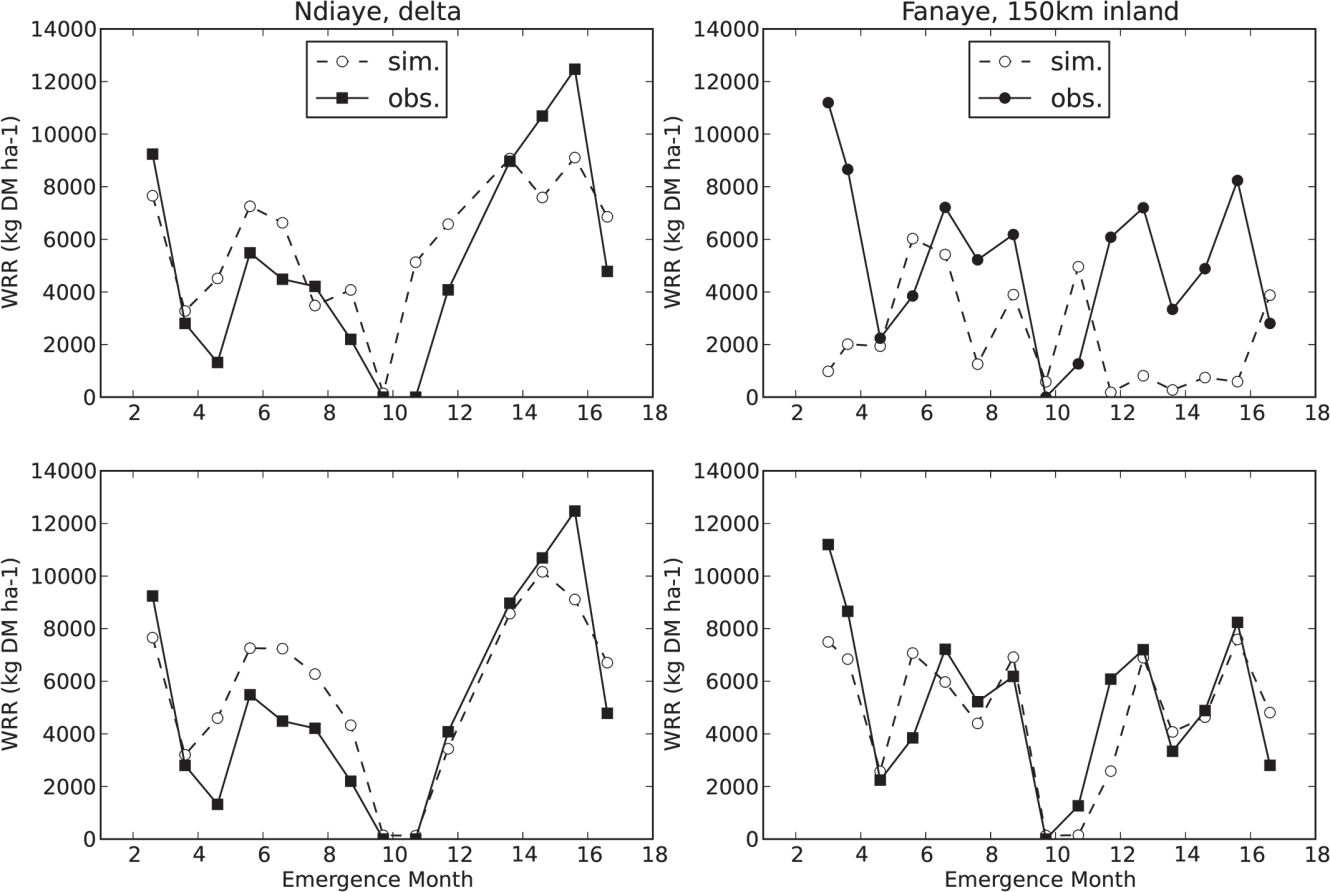
Simulated and observed yields (WRR = weight of rough rice in kilogram dry matter per hectare) for simulations with observed phenology and spikelet number. Top: with default heat and cold sterility sub-models (Table 1, s1). Bottom: with new heat and cold sterility sub-models (Table 1, s5). X-axis starts on the left (Ndiaye) mid februari 2006 and on the right (Fanaye) early march 2006. Month 14 corresponds with month 2 (February) of the next year 2007, 16 is April 2007.

**Fig 4:**
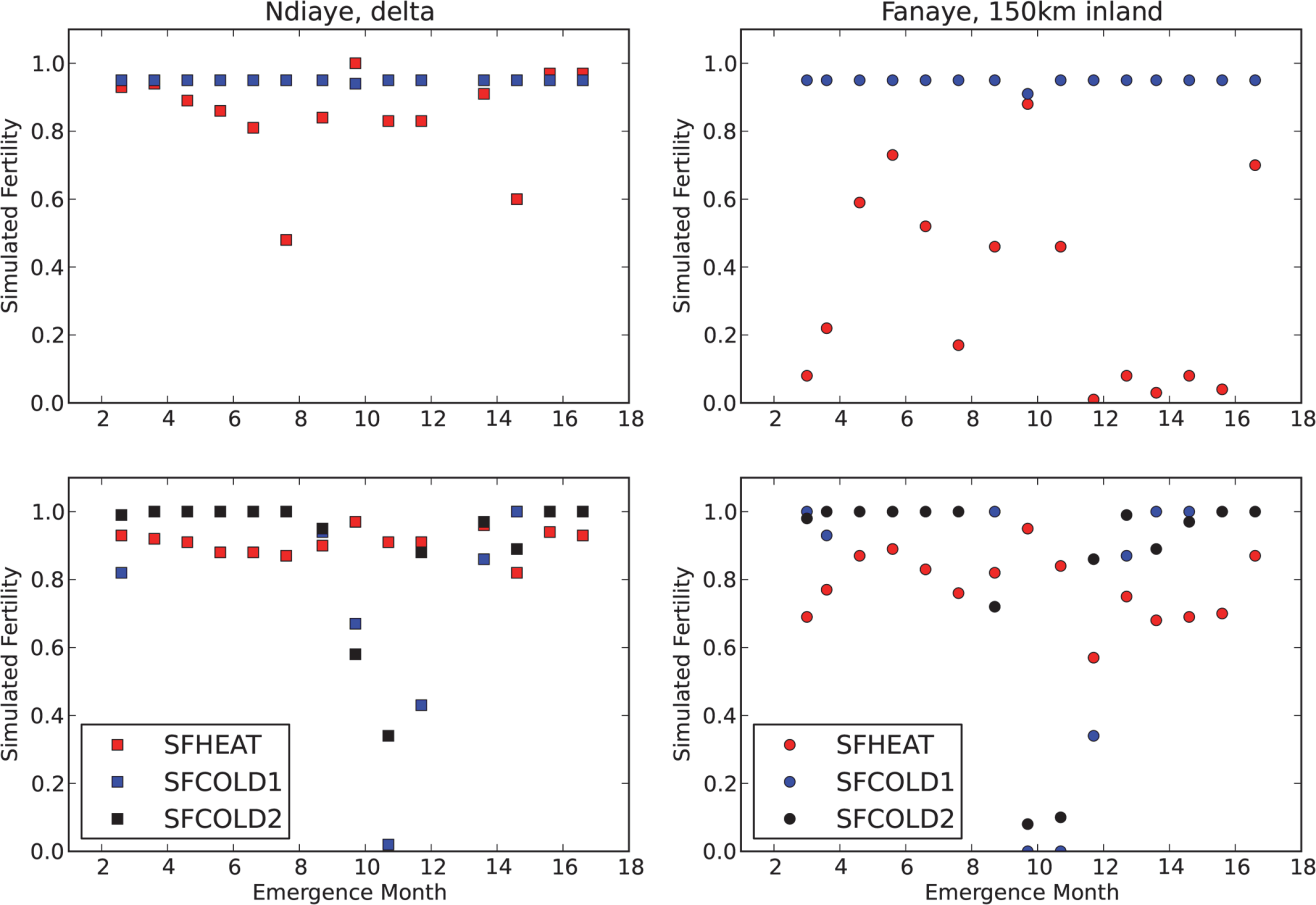
Simulated heat and cold fertility for simulations with observed phenology and spikelet number. Top: with default heat and cold sterility sub-models (Table 1, s1). Bottom: with new heat and cold sterility sub-models (Table 1, s5). X-axis starts on the left (Ndiaye) mid februari 2006 and on the right (Fanaye) early march 2006. Month 14 corresponds with month 2 (February) of the next year 2007, 16 is April 2007.

Yields were very poorly simulated when using the default heat and cold subroutines, resulting in a modelling efficiency of −0.32 (Table 1, s1), which means that predictions were worse than would be obtained by simply taking the average of observed yields. Modelling efficiency increased to 0.70 (Table 1, s5) with new heat and cold subroutines. Yields versus emergence dates with the old and new sterility models are shown in Fig. 3. The main cause of poor prediction with the default model appears to be the prediction of near zero yields on several emergence dates in Fanaye when observed yields were 4 to 11 t/ha. As a result, EF values for Fanaye are markedly lower than for Ndiaye (Table 1, s1). Fig. 4 shows in the top panes simulated old fertility and in the bottom panes new fertility. From these we can see that the large errors in Fanaye are due to gross overestimation of heat sterility. Fig. 4 also reveals large differences in simulated cold sterility. According to the default model, cold fertility is always 1, while the new cold fertility model predicts severe cold sterility for sowings in the months September (9) and October (10). Replacing the default with the new cold sterility submodel increased overall accuracy from EF 0.43 to 0.70 (s3 to s5) and increased accuracy in both sites (Fanaye: EF 0.25 →0.62, Ndiaye EF 0.55 →0.76).

**Table 1:** Simulations with observed phenology and observed spikelet number.

Model sub-version	Phenol-ogy	cold fertility	heat fertility	combined fertility	early leaf growth	partitioning to leaves	shading to kill	NSP	Modelling Efficiency (EF)			max() LAIMAX
									Fanaye + Ndiaye	Fanaye (F)	Ndiaye (N)	
s1	OBS	DEF	DEF	MIN.	DEF	DEF	NO	N/A	−0.32	−1.73	0.59	22
s2	OBS	**NEW, min.**	DEF	MIN.	DEF	DEF	NO	N/A	−0.19	−1.63	0.75	22
s3	OBS	DEF	**NEW**	MIN.	DEF	DEF	NO	N/A	0.43	0.25	0.55	22
s4	OBS	**NEW, avg.**	**NEW**	MIN.	DEF	DEF	NO	N/A	0.56	0.51	0.59	22
s5	OBS	**NEW, min.**	**NEW**	MIN.	DEF	DEF	NO	N/A	0.70	0.62	0.76	22
s6	OBS	**NEW, min.**	**NEW**	**PROD.**	DEF	DEF	NO	N/A	0.67	0.53	0.76	22
s7	OBS	**ORYZA_S**	**ORYZA_S**	**MIN.**	DEF*	**NEW**	NO	N/A	0.63	0.56	0.67	17

Phenology: observed. Cold sterility: default (Eq 22), new with minimum rule (Eq 28) or new with averaging rule (Eq 27). Heat fertility: default (Eq 14) or new (Eq 20). Combined fertility: minimum or product of heat and cold fertility. EF = modelling efficiency (Eq 32) for the two sites combined and separately for the two sites. max() LAIMAX is the maximum LAI recorded over all 30 simulations.

The minimum rule for SFCOLD (Eq 28, s5, EF = 0.70) gave more accurate simulations than the averaging rule (Eq 27, s4, EF = 0.56). The minimum rule for SPFERT gave slightly more accurate results than the product rule (s5 vs s6). We therefore proceeded with the minimum rule for both. All simulations showed unrealistically high maximum LAI values (Table 1). Additional simulations with different settings for leaf growth (not shown) had little to no effect on accuracy of yield simulations. This is somewhat surprising as we would expect assimilate supply during grain filling to be strongly affected by maximum LAI at flowering and so we would expect stronger effects on accuracy of yield simulation. The only explanation for the absence of sensitivity to pre-flowering leaf growth is that in almost all simulations the crop was sink limited. Indeed this is what we found when we compared simulated WRR and PWRR values. The comparison with ORYZA_S (s7) shows that the new model (s5) is slightly more accurate (EF 0.70 vs 0.63), in both sites. The small difference in accuracy should come as no surprise considering the shared origins of the both models and the fact that their heat and cold sterility sub-models were, at least partially, calibrated for the Sahelian environment. Although the accuracies are similar, we may hope the new ORYZA2000 subversion is more accurate than ORYZA_S when applied in more humid environments. Because the new version explicitly accounts for transpirational cooling, which will be less in humid environments, whereas ORYZA_S does not account for transpirational cooling.

### With simulated phenology and spikelet number

**Fig 5:**
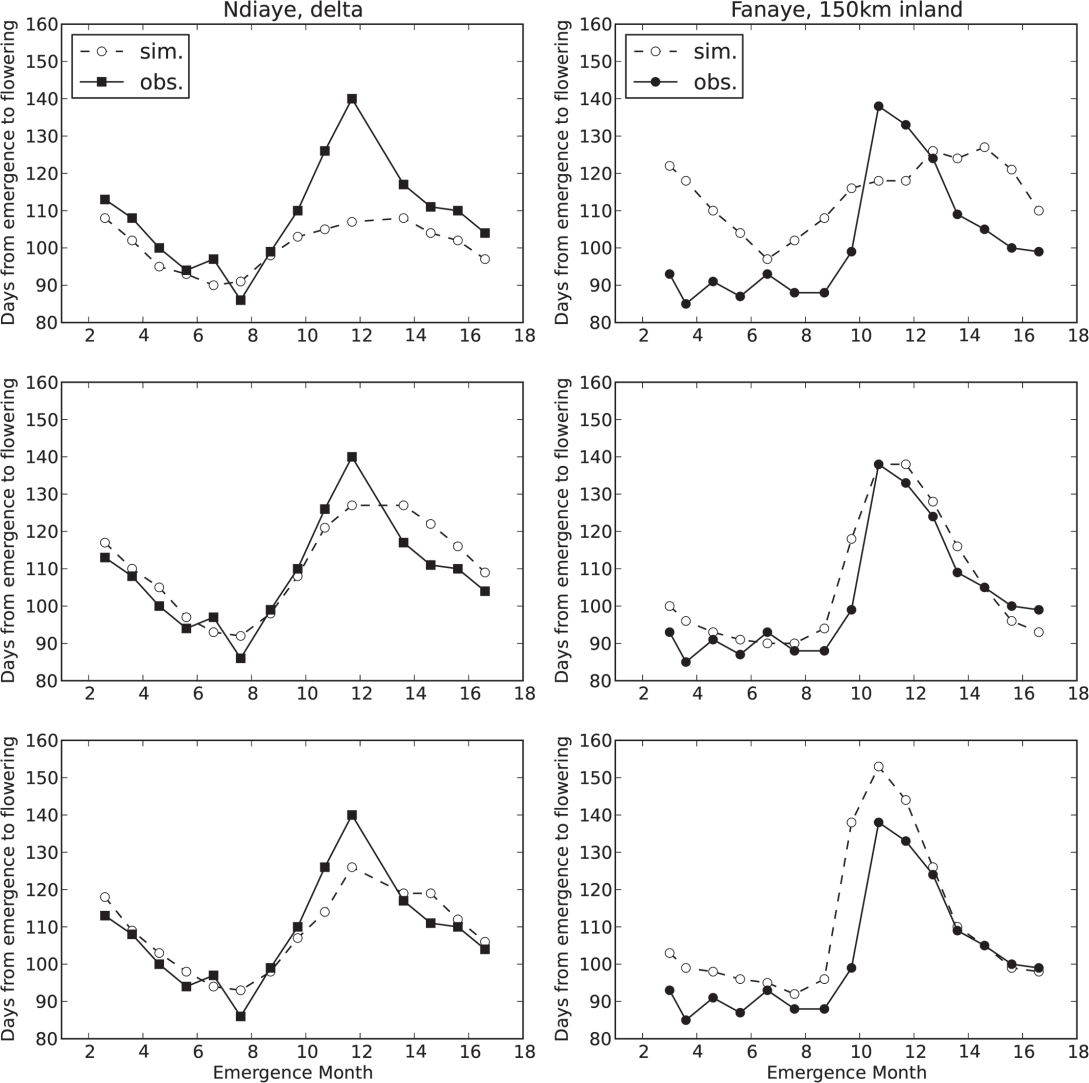
Simulated duration from emergence to flowering. Top: with default cardinal temperatures (Table 2, s8). Middle: with optimised cardinal temperatures (Table 2, s9) and Bottom: with RIDEV model (Table 2, s17). X-axis starts on the left (Ndiaye) mid februari 2006 and on the right (Fanaye) early march 2006. Month 14 corresponds with month 2 (February) of the next year 2007, 16 is April 2007.

**Fig 6:**
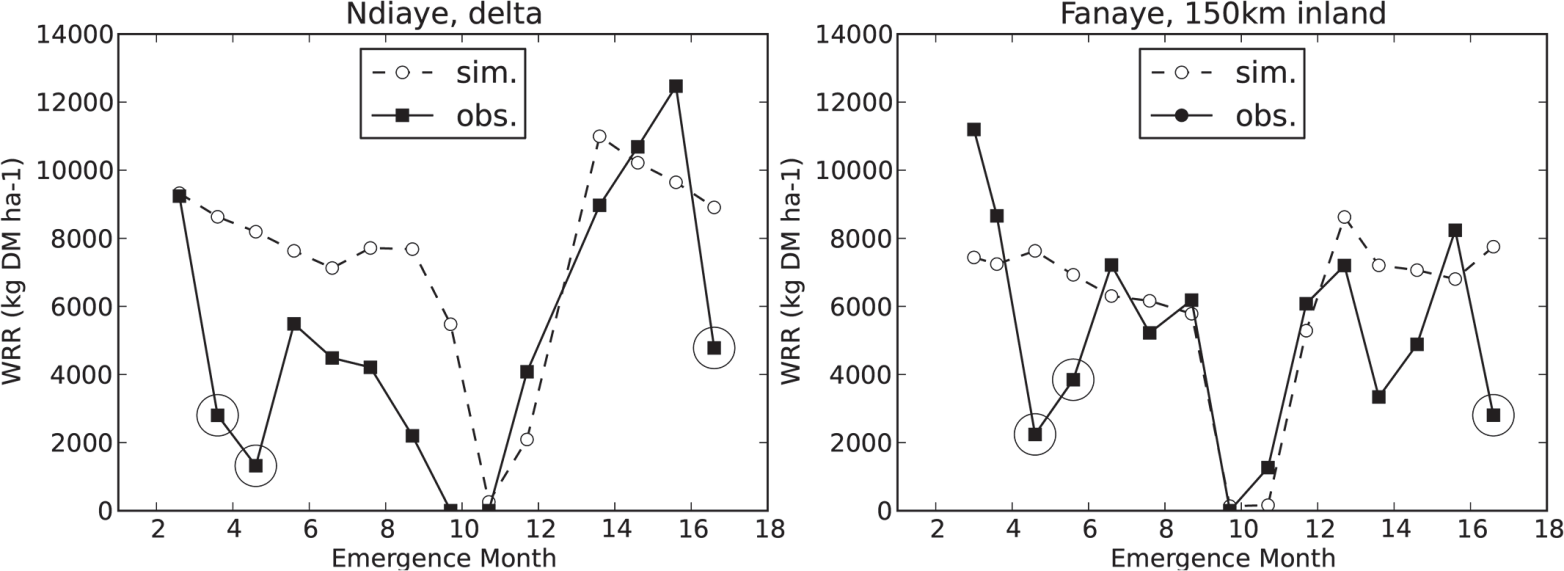
Simulated and observed yields (WRR = weight of rough rice) in Fanaye (left) and Ndiaye (right). With simulated phenology and spikelet number (Table 3 s25). WRR = weight of rough rice (kilogram dry matter per hectare). Encircled data points are outliers discussed in §3.2. X-axis starts on the left (Ndiaye) mid februari 2006 and on the right (Fanaye) early march 2006. Month 14 corresponds with month 2 (February) of the next year 2007, 16 is April 2007.

Forcing the model with observed phenology and spikelet number allowed us to inspect heat and cold sterility without being troubled by errors in biomass simulation. Now we put the model to a more severe test, by also simulating phenology (Fig. 5) and spikelet number. For six sowing dates the observed yields were much lower than expected, shown as the encircled data points in Fig. 6. We suspect that low observed yields on these dates were due to birds or other animals. Bird damage at similar dates on the same two sites was reported before by Dingkuhn and Sow [17,18] and is a known problem in the region [66]. Tables 2 and 3 show that EF values were much higher with these six outliers removed. Also striking was the fact that observed yields in Ndiaye were systematically lower than in Fanaye, while our simulations showed highest yields in Ndiaye. Previous simulations by [17,18] and observations in the same two sites [67] showed yields for Ndiaye consistently higher than yields in Fanaye. This suggests management or soil conditions were less optimal in our Ndiaye experiment, despite all efforts to avoid this. In any case, the cause of the relatively low observed yields in Ndiaye is unclear and so the issue cannot be resolved through modelling. Considering the differences between the two sites we chose to report accuracies also separately for the two sites. Given the strong evidence of improvements on the heat and cold sterility we proceeded with the new heat and cold subroutines.

**Table 2:** Simulations with modified leaf growth.

Model sub-version	Phenol-ogy	cold fertility	heat fertility	combined fertility	early leaf growth	Partitioning to leaves	shading to kill	NSP	EF Fanaye + Ndiaye	EF excl outliers			max() LAIMAX
										Fanaye + Ndiaye	Fanaye (F)	Ndiaye (N)	
s8	DEF	NEW	NEW	MIN.	DEF	DEF	NO	GCR	−0.20	0.14	−0.22	0.35	15
s9	NEW	NEW	NEW	MIN.	DEF	DEF	NO	GCR	0.01	0.41	0.49	0.36	21
s10	NEW	NEW	NEW	MIN.	**NEW**	DEF	NO	GCR	0.06	0.48	0.57	0.42	15
s11	NEW	NEW	NEW	MIN.	DEF	**NEW**	NO	GCR	0.01	0.43	0.53	0.37	18
s12	NEW	NEW	NEW	MIN.	DEF	DEF	**YES**	GCR	−0.09	0.37	0.46	0.32	13
s13	NEW	NEW	NEW	MIN.	DEF	**NEW**	**YES**	GCR	−0.09	0.39	0.48	0.34	10
s14	NEW	NEW	NEW	MIN.	**NEW**	DEF	**YES**	GCR	0.06	0.48	0.60	0.42	9
s15	NEW	NEW	NEW	MIN.	**NEW**	**NEW**	NO	GCR	0.04	0.47	0.57	0.41	13
s16	NEW	NEW	NEW	MIN.	**NEW**	**NEW**	**YES**	GCR	0.05	0.48	0.59	0.42	8
s17	**RIDEV**	**ORYZA_S**	**ORYZA_S**	MIN.	DEF*	**NEW**	NO	GCR	−0.62	−0.13	0.18	−0.31	13

Phenology: simulated with default (DEF) cardinal temperatures (8,30,42°C), simulated with new cardinal temperatures (14, 31, 999°C), or with RIDEV. Cold sterility: new with minimum rule (Eq 28). Heat fertility: new (Eq 20). Combined fertility: minimum of heat and cold fertility. Early leaf growth: simulated with default cardinal temperatures (8,30,42°C) and RGRL (0.0085oCd in ORYZA2000 (DEF) or 0.0080°Cd in ORYZA_S (DEF*)) or simulated with new cardinal temperatures (14, 31, 999°C, RGRL 0.0085°Cd). Partitioning to leaves: default as a function of development stage only or new as a function of development stage and average day temperature (Eq 7). Shading to kill: no = default, yes = leaves start to die above LAI = 4 (Eq 9). EF = modelling efficiency (Eq 32) and EF with 6 outliers with suspected bird damage removed (see Fig. 6). max() LAIMAX is the maximum LAI recorded over all 29 simulations.

**Table 3:** Simulations with modified spikelet formation.

Model sub-version	Phenology	cold fertility	heat fertility	Comb. fertility	early leaf growth	partitioning to leaves	shading to kill	NSP		EF excl outliers			
									EF Fanaye + Ndiaye	Fanaye + Ndiaye	Ndiaye + (F)	Ndiaye + (N)	max() LAIMAX
s18	NEW	NEW	NEW	MIN.	DEF	DEF	NO	GCR	0.01	0.41	0.49	0.36	21
s19	NEW	NEW	NEW	MIN.	DEF	DEF	NO	**NBIOM(Y)**	−0.20	0.17	0.51	−0.04	21
s20	NEW	NEW	NEW	MIN.	DEF	DEF	NO	**NBIOM(K)**	0.00	0.40	0.45	0.37	21
s21	NEW	NEW	NEW	MIN.	**NEW**	DEF	NO	GCR	0.06	0.48	0.57	0.42	15
s22	NEW	NEW	NEW	MIN.	**NEW**	DEF	NO	**NBIOM(Y)**	−0.06	0.34	0.57	0.20	15
s23	NEW	NEW	NEW	MIN.	**NEW**	DEF	NO	**NBIOM(K)**	0.07	0.48	0.56	0.44	15
s24	NEW	NEW	NEW	MIN.	**NEW**	DEF	**YES**	GCR	0.06	0.48	0.60	0.42	9
s25	NEW	NEW	NEW	MIN.	**NEW**	DEF	**YES**	**NBIOM(K)**	0.07	0.50	0.60	0.44	9
s26	NEW	NEW	NEW	MIN.	**NEW**	**NEW**	**YES**	**NBIOM(K)**	0.06	0.50	0.59	0.44	9

Definition of columns same as in Table 2. Additional column NSP: simulated based on GCR (Eq 10), simulated based on NBIOM (Eq 11) with (Y) Yoshida abortion function (Eq 12) or (K) Kato & Katsura abortion function (Eq 13).

First, we assessed how well the different model subversions simulated phenology. Fig. 5 shows observed and simulated number of days from emergence to flowering, simulated with ORYZA2000 default (top) and optimised (middle) cardinal temperatures and with the RIDEV model (bottom). Fig. 2 shows that the Fanaye environment is systematically hotter than Ndiaye and that crops emerging in the period from September to December will be exposed to the cool weather in the months November to March. The default cardinal temperatures overpredicted the duration from emergence to flowering in the hotter environment of Fanaye (Fig. 5 top right) and underpredicted the duration in the cooler Ndiaye (Fig. 5 top left), especially in the coolest period (emergence months 9 to 12). In an earlier paper with the same phenology and weather data van Oort et al. [22] plotted observed and simulated duration (y-axis) against average temperature during the pre-flowering phase (x-axis). These plots showed that with average temperature during the pre-flowering phase around 24°C, the default cardinal temperatures systematically underpredicted the duration by ∼20 days [22: Fig. 3A]. With average temperature during the pre-flowering phase around 31°C, the default cardinal temperatures predicted systematically a ∼20 days too long duration [22: Fig. 3A]. The RIDEV model (Fig. 4C) reproduces as similar pattern as the ORYZA2000 phenology sub model with optimised cardinal temperatures. Note that the parameters for RIDEV were determined in the early 1990s for the same variety and sites. The validation presented here shows that this model for this variety RIDEV still provides reasonable estimates of duration from emergence to flowering. We proceeded with the most accurate out of three phenology models, in this case the ORYZA2000 phenology sub model with optimised cardinal temperatures. Yield prediction was greatly improved when phenology was simulated with optimised instead of default cardinal temperatures (Table 2, s8 vs s9, EF 0.14 vs 0.41).

Next we compared, stepwise, the impact of leaf growth simulation methods on accuracy of yield simulation (Table 2, s9-s16). New (higher) cardinal temperatures for early leaf growth improved yield prediction, with overall EF increased from 0.41 to 0.48 and an increase in EF was noted in both sites. Temperature dependent partitioning and shading to kill leaves had no effect on accuracy of yield prediction. Adding these two components did lower maximum LAI to more realistic values (s10 vs s13,s14,s16). The maximum attainable modelling efficiency with revised leaf growth was 0.48 (s10, s14 and s16).

The ORYZA_S model is in terms of process functions most similar to the s9 subversion of ORYZA2000. Yet predictions with this model (Table 2, s17) were less accurate than with the newly developed subversions (s9 to s16). Since ORYZA_S differs in a number of ways from the other models it was not possible to exactly pinpoint the causes of the lower accuracy. However a visual comparison of yields over time (not shown) indicated that the main cause of the poorer accuracy was due to less accurate modelling of cold sterility.

Next we compared different methods of spikelet growth simulation (Table 3, s18-s26). Spikelet growth depends on biomass accumulation and hence on leaf area during the period from panicle initiation to flowering. Therefore we also assessed interaction with leaf growth modelling. Consistently the Yoshida et al. model led to poorer predictions than the default (GCR based) model. The Kato and Katsura model gave similar or slightly more accurate predictions than obtained with the (GCR based) default model. Both the Yoshida and the Kato and Katsura model produced a large number of juvenile spikelets (Eq 11), the Yoshida model simulated lower abortion than the Kato and Katsura model. The Yoshida model gave lower accuracies for yield simulation and this was related to overestimation of the number of mature spikelets per m^2^. Due to lack of data, it is impossible to identify the cause(s) of this overestimation. Fig. 6 shows simulated and observed yields with the most accurately simulating model subversion.

## Discussion

### Main findings

Our research integrates insights from recent experimental work into an improved subversion of the ORYZA2000 model. The main findings are:

1. The default model overestimates heat sterility and underestimates cold sterility in arid environments.
2. Yield prediction was greatly improved with the new heat and cold subroutines.
3. The key mechanisms of rice to avoid heat sterility are transpirational cooling and early morning flowering. These adaptive mechanisms allow rice to be grown in arid environments with extreme heat (provided that there are enough water and nutrients).
4. The default model overpredicted the length of growing seasons at high temperatures and underpredicted the length of growing seasons at lower temperatures, for the variety considered here (IR64).
5. Use of different cardinal temperatures, in particular a higher base temperature and higher maximum temperature, improved phenology simulation and this in turn lead to improved yield simulation.
6. Although LAI development could not be compared with observed values, the yield simulations were improved when for early leaf growth higher cardinal temperatures than default were used
7. New methods for simulation of spikelet formation led to little or no increase in accuracy of yield simulation.
8. The adapted subversion of ORYZA2000 was more accurate than a previously developed model for rice in the same environments (ORYZA_S).

The model was validated with a relatively large dataset in an arid environment and a wide range of temperatures. Additional validations in other environments are still needed. We expect that with the incorporation of environment dependent transpirational cooling and early morning flowering and with cold sterility simulated with minimum rather than average temperature, the adapted subversion of ORYZA2000 presented in this paper will be more robust than its predecessors which did not account for these processes.

### Interpretation of the results

A model is always a simplification of reality. Perfect prediction is suspect, may be caused by over parameterisation on a limited dataset and runs a risk of adjusting parameter values without sound ecophysiological justification. We have tried to avoid this by using a large dataset, by making only modifications substantiated by solid experimental research and by keeping calibration to a minimum. Only phenological parameters were calibrated. In our experiments we were confronted with missing data and practical difficulties in realising potential production. It is interesting to see that despite these uncertainties and with a minimum of calibration, the adapted model subversions predicted yields reasonably well. The systematic stepwise incorporation of new model components allowed for assessing the effect of different individual and combined changes in the model on accuracy of yield simulation. Some modifications did contribute to improved yield simulation (heat and cold sterility; cardinal temperatures for phenology; cardinal temperatures for early leaf growth). Other modifications did not contribute to improved yield simulation (temperature dependent assimilate partitioning to leaves; shading effects on leaf death; spikelet formation).

This study is not a new validation of individual processes (phenology, early leaf growth, assimilate partitioning, leaf senescence, spikelet formation, heat sterility, cold sterility). For phenology, calibration by van Oort et al. [22] was on the same dataset as used in this paper. For the other processes: we did not measure leaf area during growth, biomass growth and nitrogen from PI to flowering and pollination, so we could not directly test the model components evaluated here. What we did was asking the question “suppose that this is a better description of reality for a processes at level n-1, then does this help us better predict yield? (an emerging property at level n)”. If so, it adds credibility, but not proof, to the new process description. The results call for further research into the model components addressed in this paper. For example, we propose dedicated experiments on LAI growth at different sowing dates and in different environments to estimate the early growth parameters (TBD, TOD, TMD, RGRL) instead of simulating them as we did in this paper by assuming the same cardinal temperatures as obtained for phenology and using the default value for RGRL. The fact that modifications such as shading to kill leaves and new methods of spikelet formation did not lead to increased accuracy in yield simulation does not invalidate these processes. Possibly if fed with observed crop nitrogen contents in the current study, or in other environments for example with less nitrogen, the Yoshida et al. [27] and Kato and Katsura [28] models would lead to better predictions than the default model. Such hypotheses can be tested more easily now that these sub-models have been incorporated in a larger model. In summary, the modelling work presented here has generated new hypotheses that can be subsequently tested experimentally. This paper has shown the relevance of testing these hypotheses for increased accuracy of yield prediction.

As we noted in the introduction, the default ORYZA2000 model has been calibrated and validated in many environments before, with high accuracies. How is this possible considering the poor performance reported here? There are a number of underlying reasons why issues raised in this paper were not previously detected. Firstly, the arid environment considered here is quite uncommon in Asian countries where ORYZA2000 has mostly been used. As we discussed (and shown before in [17,18]) the arid climate creates some typical conditions affecting heat sterility (strong transpirational cooling) and cold sterility (dangerously low night temperature, acceptable average temperature, due to large diurnal temperature amplitude). The model has rarely before been tested in environments as extreme as considered in this paper and it is only in these extreme environments that errors in the phenology and sterility models become apparent. Secondly the sowing dates considered here were far off from the optimum, exposing the crop to severe cold sterility. Normally in agronomic experiments one would never sow in some of the sowing dates considered in this paper. Then normally also the failures of the model under such cold conditions would not be discovered. They would only be detected in experiments dedicated to impose cold stress. Thirdly, the conditions under which extremely high LAI were simulated are exceptional: low temperatures during the vegetative stage leading to a long duration from emergence to flowering (up to 130–140 days) combined with high radiation levels and high daytime temperatures during this period allowing for high leaf biomass accumulation. Fourthly, acknowledging that any model is a simplification of reality Bouman et al. [8: p. 196] identified those parts of the model which they thought to be less generic, requiring additional calibration specifically for the variety and environment at hand. This list amongst others includes the development rates and the relative leaf growth rate. Possibly the issue of the validity of the cardinal temperatures was never detected because people were resolving the issue through calibration of the rates. Especially when calibrating separately for different environments and varieties, or when calibrating for a limited number of experiments, errors in cardinal temperatures may remain undetected. The interesting question is whether more generic, variety specific development rates and relative leaf growth rates can be found once we recalculate them with more appropriate cardinal temperatures. It seems unlikely that there would exist one unique set of cardinal temperatures valid for all rice varieties; genetic variation is simply too large for this [22,43,44]. What we hypothesise is that once a set of cardinal temperatures has been appropriately estimated for a particular variety, recalibration of development rates and relative leaf growth will not be necessary every time again when the model is applied in new environments. So that parameters become truly genetic parameters, not needing to be recalibrated when applied in different environments. Large datasets for the same varieties in contrasting environments are needed to test the hypothesis that more generically valid crop parameters exist.

### Implications

In the previous section we highlighted how modelling exercises such as presented in this paper can be used to increase insights in a particular system and to narrow knowledge gaps. A second more common role for modelling is in decision support. Models can be used in this role if they are sufficiently accurate. In ideotyping, genetic parameters are changed and it is assessed whether these lead to desirable changes such as crop yield [68], in some cases also tailored to specific environments [69]. Models can also be of use for investigating different management options, such as irrigation scheduling and amount, fertilisation scheduling and amount, effects of bund height, etc [5,11,12,13,14,70]. Models can be used to simulate if it is more economical to growth one or two crops per year and for deriving the optimum sowing dates for different sites [17,18]. At the farm level, models can be used to investigate economically optimal water allocation strategies, dividing available water among different crops [21]. Models can be used for climate change impact studies and can in this role help shape the research agenda into adaptation strategies [1,2]. All such model applications are contingent on the accuracy of the model. The most thorough and wide spanning climate change impact assessment in rice was by Matthews et al. [1,2] for Asia. In different parts of Asia negative effects of climate change were predicted with the ORYZA2000 and SIMRIW models which used the same heat sterility subroutines. Analyses of yield declines identified heat sterility as the main cause of yield declines in Southern Japan, India, South Korea and China. Other studies [24,26,71] report temperature thresholds similar to those used in the default ORYZA2000 model. Such temperature thresholds have recently been used in global climate change assessments [3,4]. The recent studies on rice adaptive mechanisms to heat stress [19,20,29,30,32] and the findings of this study call for a re-assessment of climate risks for rice production, in the current and future climate. It is known that genotypic differences exist in cardinal temperatures, heat tolerance and heat avoidance. Selection for appropriate varieties adapted to their climate is therefore possible.

## Conclusions

ORYZA2000 could not well simulate yields in two arid environments. New research on several model components was systematically and stepwise incorporated into the existing model and effects on increased accuracy were investigated. The most important finding is that in arid environments the model overestimates heat sterility and underestimates cold sterility. The cause for overestimation of heat sterility was that the default model ignored early morning flowering and transpirational cooling. The cause for underestimating cold sterility was that the default model calculated with daily average rather than daily minimum temperatures. Other model improvements were on cardinal temperatures for phenology [22] and cardinal temperatures for early leaf growth. Alternative models for simulation of spikelet formation did not lead to improved yield prediction.

## Supporting Information

S1 Table List of variables and parameters. (DOCX)

## References

[1] Matthews RB, Kropff MJ, Bachelet D, van Laar HH (eds) (1995). Modeling the Impact of Climate Change on Rice Production in Asia. IRRI/CAB International.

[2] Matthews RB, Kropff MJ, Horie T, Bachelet D (1995) Simulating the Impact of Climate Change on Rice Production in Asia and Evaluating Options for Adaptation. Agric Syst. 54(3): 399–425.

[3] Gourdji SM, Sibley AM, Lobell DB. Global crop exposure to critical high temperatures in the reproductive period: Historical trends and future projections. Environ Res Lett. 8(2): 24–41.

[4] Teixeira EI, Fischer G, Van Velthuizen H, Walter C, Ewert F (2013) Global hot-spots of heat stress on agricultural crops due to climate change. Agric For Meteorol. 170: 206–215.

[5] Belder P, Bouman BAM, Spiertz JHJ (2007) Exploring options for water savings in lowland rice using a modelling approach. Agric Syst. 92(1–3): 91–114.

[6] Boling AA, Bouman BAM, Tuong TP, Konboon Y, Harnpichitvitaya D (2011) Yield gap analysis and the effect of nitrogen and water on photoperiod-sensitive jasmine rice in north-east Thailand. Neth J Agr Sci 58: 11–19.

[7] Boling AA, Bouman BAM, Tuong TP, Murty MVR, Jatmiko SY (2007) Modeling the effect of groundwater depth on yield-increasing interventions in rainfed lowland rice in Central Java, Indonesia. Agric Syst. 92(1–3): 115–139.

[8] Bouman BAM, Kropff MJ, Tuong TP, Wopereis MCS, ten Berge HFM, et al. (2001) ORYZA2000: Modeling Lowland Rice. International Rice Research Institute, Los Baños, Philippines and Wageningen University and Research Centre, Wageningen, The Netherlands.

[9] Bouman BAM, van Laar HH (2006) Description and evaluation of the rice growth model ORYZA2000 under nitrogen-limited conditions. Agric Syst. 87(3): 249–273.

[10] Bouman BAM, Feng L, Tuong TP, Lu G, Wang H, Feng Y (2007) Exploring options to grow rice under water-short conditions in northern China using a modelling approach. II: quantifying yield, water balance components, and water productivity. Agric Water Manage. 88: 23–33.

[11] Feng L, Bouman BAM, Tuong TP, Cabangon RJ, Li Y, Lu G, Feng Y (2007) Exploring options to grow rice under water-short conditions in northern China using a modelling approach. I: Field experiments and model evaluation. Agric Water Manage. 88(1–3): 1–13.

[12] Jing Q, Bouman BAM, Hengsdijk H, Van Keulen H, Cao W (2007) Exploring options to combine high yields with high nitrogen use efficiencies in irrigated rice in China. Eur J Agron. 26(2): 166–177.

[13] Jing Q, Bouman BAM, Van Keulen H, Hengsdijk H, Cao W, et al (2008) Disentangling the effect of environmental factors on yield and nitrogen uptake of irrigated rice in Asia. Agric Syst. 98(3): 177–188.

[14] Soundharajan B, Sudheer KP (2009) Deficit irrigation management for rice using crop growth simulation model in an optimization framework. Paddy and Water Environ. 7(2): 135–149.

[15] Li T, Raman AK, Marcaida M, Kumar A, Angeles O, et al (2013) Simulation of genotype performances across a larger number of environments for rice breeding using ORYZA2000. Field Crops Res. 149(): 312–321.

[16] Kropff MJ, van Laar HH, ten Berge HFM (1993) ORYZA1, a basic model for irrigated lowland rice SARP report, International Rice Research Institute, Manila, Philippines.

[17] Dingkuhn M, Sow A (1997) Potential yields of irrigated rice in the Sahel In: Miézan KM, Wopereis MCS, Dingkuhn M, Deckers J, Randolph TF (Eds.), Irrigated rice in the Sahel: Prospects for sustainable development. WARDA, ADRAO, Bouaké, Côte d'Ivoire; pp. 361–379.

[18] Dingkuhn M, Sow A (1997) Potential yields of irrigated rice in arid environments In: Kropff MJ, Teng P, Aggarwal PK, Bouma J, Bouman BAM, Jones JW, Van Laar HH (Eds.). Applications of Systems Approaches at the Field Level Volume 2: Proceedings of the Second International Symposium on Systems Approaches for Agricultural Development, held at IRRI, Los Baños, Philippines, 6–8 December 1995, pp. 79–100.

[19] Julia C, Dingkuhn M (2012) Variation in time of day of anthesis in rice in different climatic environments. Eur J Agron. 43: 166–174.

[20] Julia C, Dingkuhn M (2013) Predicting temperature induced sterility of rice spikelets requires simulation of crop-generated microclimate. Eur J Agron. 49: 50–60.

[21] Gaydon DS, Meinke H, Rodriguez D, McGrath DJ (2012) Comparing water options for irrigation farmers using Modern Portfolio Theory. Agric Water Manage. 115: 1–9.

[22] van Oort PAJ, Zhang T, de Vries ME, Heinemann AB, Meinke H (2011) Correlation between temperature and phenology prediction error in rice (Oryza sativa L.). Agric For Meteorol. 151(12): 1545–1555.

[23] Nishiyama I (1976) Effect of temperature on the vegetative growth of rice In: “Climate and Rice, International Rice Research Institute, Los Banos, Phillipines; pp. 159–186.

[24] Yoshida S (1981) Fundamentals of Rice Crop Science. International Rice Research Institute, Los Banos, Philippines.

[25] Rebolledo MC, Dingkuhn M, Péré P, Mcnally KL, Luquet D (2012) Developmental Dynamics and Early Growth Vigour in Rice. I. Relationship Between Development Rate (1/Phyllochron) and Growth. J Agron. Crop Sci. 198(5): 374–384.

[26] Sánchez B, Rasmussen A, Porter JR (2014) Temperatures and the growth and development of maize and rice: A review. Glob Change Biol. 20(2): 408–417. doi: 10.1111/gcb.12389 24038930 10.1111/gcb.12389

[27] Yoshida H, Horie T, Shiraiwa T (2006) A model explaining genotypic and environmental variation of rice spikelet number per unit area measured by cross-locational experiments in Asia. Field Crops Res. 97 (2–3): 337–343.

[28] Kato Y, Katsura K (2010) Panicle architecture and grain number in irrigated rice, grown under different water management regimes. Field Crops Res. 117(2–3): 237–244.

[29] Ishimaru T, Hirabayashi H, Ida M, Takai T, San-Oh YA, Yoshinaga S, et al (2010) A genetic resource for early-morning flowering trait of wild rice Oryza officinalis to mitigate high temperature-induced spikelet sterility at anthesis. Ann Bot. 106(3): 515–520. doi: 10.1093/aob/mcq124 20566680 10.1093/aob/mcq124 PMC2924824

[30] Kobayasi K, Matsui T, Yoshimoto M, Hasegawa T (2010) Effects of temperature solar radiation, and vapor-pressure deficit on flower opening time in rice. Plant Prod Sci. 13: 21–28.

[31] Matsui T, Kobayasi K, Yoshimoto M, Hasegawa T (2007) Stability of rice pollination in the field under hot and dry conditions in the Riverina Region of New South Wales, Australia. Plant Prod Sci. 10: 57–63.

[32] van Oort PAJ, Saito K, Swart SJ, Shrestha S. (2014) A simple model for simulating rice heat sterility as a function of flowering time and transpirational cooling. Field Crops Res. 156: 303–312.

[33] Yoshimoto M, Fukuoka M, Hasegawa T, Utsumi M, Ishigooka Y, et al (2011) Integrated micrometeorology model for panicle and canopy temperature (IM2PACT) for rice heat stress studies under climate change. J Agr Meteorol. 67: 233–247.

[34] Yoshimoto M, Oue H, Takahashi H, Kobayashi K (2005) The effects of FACE (Free-Air CO2 Enrichment) on temperatures and transpiration of rice panicles at flowering stage. J Agr Meteorol. 60: 597–600.

[35] Uchijima T (1976) Some aspects of the relation between low air temperature and sterile spikelets numbers in rice plants. (In Japanese.). Jpn J Agric Meteorol. 31:199–202.

[36] Horie T. (1993) Predicting the effect of climate variation and elevated CO2 on rice yield in Japan. Jpn J Agric Meteorol. 48: 567–574.

[37] Farrell TC, Fox KM, Williams RL, Fukai S (2006) Genotypic variation for cold tolerance during reproductive development in rice: screening with cold air and cold water. Field Crops Res. 98: 178–194.

[38] Shimono H, Okada M, Kanda E, Arakawa I (2007) Low temperature-induced sterility in rice: Evidence for the effects of temperature before panicle initiation. Field Crops Res. 101(2): 221–231.

[39] Dingkuhn M, Julia C, Soulie JC (2012) Development of RIDEV V.2 Rice Model of Phenology and Thermal Sterility of Spikelets. Terminal Report (preliminary version). CIRAD/IRRI, 07/04/2012

[40] Julia C (2012) Thermal stresses and spikelet sterility in rice: sensitive phases and role of microclimate. PhD thesis University of Montpellier.

[41] Confalonieri R, Mariani L., Bocchi S. (2005) Analysis and modelling of water and near water temperatures in flooded rice (Oryza sativa L.). Ecol. Modell. 183: 269–280.

[42] Kuwagata T, Hamasaki T, Watanabe T (2008) Modeling water temperature in a rice paddy for agro-environmental research. Agric For Meteorol. 148: 1754–1766.

[43] Yin X, Kropff MJ, Horie T, Nakagawa H, Centeno HGS, Zhu D, Goudriaan J (1997) A model for photothermal responses of flowering in rice. I. Model description and parameterization. Field Crops Res. 51: 189–200.

[44] Yin X, Kropff MJ, McLaren G, Visperas RM (1995) A nonlinear model for crop development as a function of temperature. Agric. Forest Meteorol. 77, 1–16.

[45] Zhang S, Tao F (2013) Modeling the response of rice phenology to climate change and variability in different climatic zones: Comparisons of five models. Eur J Agron. 45: 165–176.

[46] Spitters CJT, van Keulen H, van Kraalingen DWG (1995) A simple and universal crop growth simulator: SUCROS87 In: Rabbinge R, Ward SA, van Laar HH (Eds.) Simulation and systems management in crop protection. Simulation Monographs, Pudoc, Wageningen, The Netherlands.

[47] Van Laar HH, Goudrian J, van Keulen H (1997) SUCROS97: Simulation of potential and water-limited production situations. Quantitative Approaches in Systems Analysis no. 14, Wageningen, The Netherlands.

[48] Horie T (2001) Increasing yield potential in irrigated rice: breaking the barrier In Peng S. and Hardy B. eds.Rice Research for Food Security and Poverty Alleviation. Proc. Int. Rice Res. Conf., 31 Mar.–3 Apr. 2000, Los Baños, Philippines, IRRI; 2001.

[49] Sheehy JE, Dionora MJA, Mitchell PL (2001) Spikelet numbers, sink size and potential yield in rice. Field Crops Res. 71(2): 77–85.

[50] Jagadish SVK, Craufurd PQ, Wheeler TR (2007) High temperature stress and spikelet fertility in rice (Oryza sativa L.). J Exp Bot. 58 (7): 1627–1635. 17431025 10.1093/jxb/erm003

[51] Weerakoon WMW, Maruyama A, Ohba K (2008) Impact of humidity on temperature-induced grain sterility in rice (Oryza sativa L). J Agron Crop Sci. 194(2): 135–140.

[52] Goudriaan J, van Laar HH (1994) Modelling potential crop growth processes Current Issues in Production Ecology, Kluwer Academic Publishers, Dordrecht, The Netherlands.

[53] Ephrath JE, Goudriaan J, Marani A (1996) Modelling diurnal patterns of air temperature, radiation wind speed and relative humidity by equations from daily characteristics. Agric Syst. 51(4): 377–393.

[54] Matsui T, Omasa K, Horie T (1997) High temperature-induced spikelet sterility of Japonica rice at flowering in relation to air temperature, humidity and wind velocity conditions. Jpn J Crop Sci. 66: 449–455.

[55] Matsui T, Omasa K (2002) Rice (Oryza sativa L.) Cultivars tolerant to high temperature at flowering: Anther characteristics. Ann Bot. 89(6): 683–687. 12102523 10.1093/aob/mcf112 PMC4233832

[56] Matsui T, Omasa K, Horie T (1999) Mechanism of anther dehiscence in rice (Oryza sativa L.). Ann Bot. 84(4): 501–506.

[57] Matsui T, Omasa K, Horie T (2000) High temperature at flowering inhibits swelling of pollen grains, a driving force for thecae dehiscence in rice (Oryza sativa L.). Plant Prod Sci. 3(4): 430–434.

[58] Jagadish SVK, Craufurd PQ, Wheeler TR (2008) Phenotyping parents of mapping populations of rice for heat tolerance during anthesis. Crop Sci. 48(3): 1140–1146.

[59] de Vries ME, Leffelaar PA, Sakan É N, Bado BV, Giller KE (2011) Adaptability of irrigated rice to temperature change in Sahelian environments. Exp Agric. 47(1): 69–87.

[60] Dingkuhn M, Sow A, Samb A, Diack S, Asch F (1995) Climatic determinants of irrigated rice performance in the Sahel—I. Photothermal and micro-climatic responses of flowering. Agric Syst. 48(4): 385–410.

[61] Kobata T, Akiyama Y, Kawaoka T (2010) Convenient Estimation of Unfertilized Grains in Rice. Plant Prod Sci. 13(3): 289–296.

[62] Jagadish KSV, Cairns JE, Kumar A, Somayanda IM, Craufurd PQ (2011) Does susceptibility to heat stress confound screening for drought tolerance in rice? Funct Plant Biol. 38(4): 261–269. 10.1071/FP10224 32480882

[63] White JW, Hoogenboom G, Wilkens PW, Stackhouse PW Jr., Hoel JM (2011) Evaluation of satellite-based, modeled-derived daily solar radiation data for the continental United States. Agron J. 103: 1242–1251.

[64] Nash JE, Sutcliffe JV (1970) River flow forecasting through conceptual models part I—A discussion of principles. J Hydrology 10(3): 282–290.

[65] de Vries ME, Rodenburg J, Bado BV, Sow A, Leffelaar PA, et al (2010) Rice production with less water is possible in a Sahelian environment. Field Crops Res. 116: 154–164.

[66] de Mey Y, Demont M, Diagne M (2012) Estimating Bird Damage to Rice in Africa: Evidence from the Senegal River Valley. J Agr Econ. 63(1): 175–200.

[67] Haefele SM, Wopereis MCS, Wiechmann H (2002) Long-term fertility experiments for irrigated rice in the West African Sahel: agronomic results. Field Crops Res. 78(2–3): 119–131.

[68] Peng S, Khush GS, Virk P, Tang Q, Zou Y (2008) Progress in ideotype breeding to increase rice yield potential. Field Crops Res. 108(1): 32–38.

[69] Heinemann AB, Dingkuhn M, Luquet D, Combres JC, Chapman S (2008) Characterization of drought stress environments for upland rice and maize in central Brazil. Euphytica 162(3): 395–410.

[70] ten Berge HFM, Thiyagarajan TM, Shi QH, Wopereis MCS, Drenth H, et al (1997) Numerical optimization of nitrogen application to rice. 1. Description of MANAGE-N. Field Crops Res. 51(1–2): 29–42.

[71] Wassmann R, Jagadish SVK., Heuer S, Ismail A, Redona E, et al (2009) Chapter 2 Climate Change Affecting Rice Production. The Physiological and Agronomic Basis for Possible Adaptation Strategies. Adv Agron. 101: 59–122.

