# Supplementary material for: Improved Climate Risk Simulations for Rice in Arid Environments
Source: PLoS One. 2015 Mar 16;10(3):e0118114. doi: 10.1371/journal.pone.0118114 (PMC4361630; doi:10.1371/journal.pone.0118114)
Supplement: S1 Table — (DOCX) [file pone.0118114.s001.docx]

**S1 Table List of variables and parameters**

| Acronym | Unit | Description | Equation |
| --- | --- | --- | --- |
| GCR | kg DM ha^-1^ d^-1^ | Gross growth rate of crop (DM = dry matter) | 10 |
| GCR2BF | kg DM ha^-1^ | Gross dry matter growth of crop from DVS 0.825 to 1.0 (DM = dry matter) | 12,13 |
| CNT2BF | d | Number of days from DVS 0.825 to 1.0 | 12 |
| COLDTT | ^o^Cd | Accumulated cold degree days | 21 |
| DL | h | Day length | 16 |
| DVR_BVP_ | d^-1^ | Development rate in the basic vegetative phase | 1 |
| DVR_GFP_ | d^-1^ | Development rate in the grain filling phase | 3 |
| DVR_PPP_ | d^-1^ | Development rate in the post PSP phase | 2 |
| DVR_PSP_ | d^-1^ | Development rate in the photoperiod sensitive phase | 1 |
| DVS |  | Development stage | 7 |
| EF |  | Modelling Efficiency | 32 |
| FFS |  | Fraction filled spikelets | text |
| FLV |  | Fraction of assimilates partitioned to leaves | 7 |
| GLAI | m^2^ leaf m^-2^ soil d^-1^ | Growth in LAI per day | 6 |
| HULV | ^o^Cd | Daily heat units for leaf growth | 6 |
| HULV(t) | ^o^Ch | Hourly or daily heat units for leaf growth at time t (0-24h) | 5 |
| LAI | m^2^ leaf m^-2^ soil | Leaf area index | 6,9,23 |
| LAICR | m^2^ leaf m^-2^ soil | Critical LAI, if LAI > LAICR then leaves start dying due to shading | 9 |
| LLVSH | kg DM ha^-1^ d^-1^ | Loss of leaf dry matter due to shading (DM = dry matter) | 9 |
| LTR |  | Light Transmission Ratio | 23 |
| NBIOM | kg N ha^-1^ | Total N of the aboveground biomass | 11,12,13 |
| NSP | no. ha^-1^ | Number of spikelets | 10,12,13,30 |
| NSPJUV | no. ha^-1^ | Number of juvenile spikelets | 11,12,13 |
| OBSDUR_BVP_ | d | Observed duration of the basic vegetative phase | 1 |
| OBSDUR_GFP_ | d | Observed duration of the grain filling phase | 3 |
| OBSDUR_PPP_ | d | Observed duration of the post PSP phase | 2 |
| OBSDUR_PSP_ | D | Observed duration of the photoperiod sensitive phase | 1 |
| PWRR | kg DM ha^-1^ | Potential weight of rough rice (DM = dry matter) | 31 |
| RGRL | ^o^Cd^-1^ | Relative growth rate of the leaves | 6 |
| SFCOLD |  | Cold fertility | 22 |
| SFCOLD1 |  | Cold fertility in the microspore development phase, on a specific day | 25,26,27,28 |
| $\bar{\text{SFCOLD1}}$ |  | Cold fertility aggregated over duration of the microspore development phase | 27,28 |
| SFCOLD2 |  | Cold fertility in the panicle exsertion development phase, on a specific day | 29,30 |
| $\bar{\text{SFCOLD2}}$ |  | Cold fertility aggregated over duration of the panicle exsertion development phase | 30 |
| SFHEAT(t) |  | Heat fertility on a given day when flowering occurs at time t (0-24h) | 19 |
| $\bar{\text{SFHEAT}}$ |  | Heat fertility, aggregated over the period in which sensitivity to heat sterility occurs (DVS 0.96-1.2) | 14,20 |
| SIMDUR_DVS1-DVS2_ | d | Simulated number of days from development stage DVS1 to DVS2 | 20,27 |
| SPFERT |  | Combined fertility | text |
| SPGF | no. kg^-1^ | Spikelet growth factor | 10 |
| SPGFJ | no. kg^-1^ | Spikelet growth factor for juvenile spikelets | 11 |
| t | h | Time (0-24h) | 4,5 |
| T_air_(t) | ^o^C | Air temperature at 2 meters height in an open field at time t (0-24h) | 4,5,16 |
| TAV | ^o^C | Daily average temperature | 7,8,21 |
| TBD | ^o^C | Base temperature for development | Text |
| TBLV | ^o^C | Base temperature for early leaf growth | 5 |
| T_m,a_ | ^o^C | Average of maximum air temperatures from DVS 0.96 to 1.2 | 14 |
| T_max_ |  | Maximum temperature of the day | 4,7 |
| TMD | ^o^C | Maximum temperature for development & leaf growth | 5 |
| T_min_ | ^o^C | Minimum temperature of the day | 4,7 |
| T_min7_ | ^o^C | Average of minimum temperatures in the preceding 7 days | 16 |
| TOD | ^o^C | Optimum temperature for development & leaf growth | 5 |
| t_peakfl_ | h | Peak flowering time | 15 |
| t_sunrise_ | h | Time of sunrise | 15 |
| T_w,min_ | ^o^C | Minimum floodwater temperature | 24 |
| VPA | kPa | Early morning vapour pressure | 17 |
| VPD(t) | kPa | Vapour pressure deficit at time t (0-24h) | 17 |
| WGRMX | kg DM grain^-1^ | Maximum individual grain weight (DM = dry matter) | 31 |
| WLVG | kg DM ha^-1^ | Dry weight of green (= alive) leaves (DM = dry matter) | 9 |
| WRR | kg DM ha^-1^ | Weight of rough rice (DM = dry matter) | text |
